# Optimal error estimates of the diffuse domain method for second order parabolic equations

**DOI:** 10.1007/s10543-026-01118-8

**Published:** 2026-03-30

**Authors:** Wenrui Hao, Lili Ju, Yuejin Xu

**Affiliations:** 1 Department of mathematics, Pennsylvania State University, College Park, 16802 PA USA; 2 Department of Mathematics, University of South Carolina, Columbia, 29208 USA

**Keywords:** Parabolic equations, Irregular domains, Diffuse domain method, Weighted norms, Error estimates, 65M15, 65M60, 65Y20

## Abstract

In this paper, we study the convergence behavior of the diffuse domain method (DDM) for solving a class of second-order parabolic partial differential equations with Neumann boundary condition posed on general irregular domains. The DDM employs a phase-field function to extend the original parabolic problem to a similar but slightly modified problem defined over a larger rectangular domain that contains the target physical domain. Based on the weighted Sobolev spaces, we rigorously establish the convergence of the diffuse domain solution to the original solution as the interface thickness parameter goes to zero, together with the corresponding optimal error estimates under the weighted $L^2$ and $H^1$ norms. Numerical experiments are also presented to validate the theoretical results.

## Introduction

Combined with appropriate initial values and boundary conditions, parabolic partial differential equations (PDEs) have been widely applied in various mathematical models. Notable examples include the Navier-Stokes equations and the time-dependent advection-diffusion equation in fluid dynamics [24], Stokes-Darcy problems arising in petroleum and biomedical engineering [13], as well as the Allen-Cahn equation and other phase field models used to describe phase transitions and separations [5, 18], among many others. Most existing numerical methods for solving interface problems are based on sharp interface approaches, which rely on explicit surface parameterization. This requirement poses a significant challenge for handling complex geometries and mesh generation. Examples of such methods include the extended and composite finite element methods [17, 22], immersed interface methods [31, 34, 43], virtual node methods with embedded boundary conditions [8, 26], and matched interface and boundary methods [7, 32, 45], among others. Many of these techniques require specialized computational tools that are not readily available in standard finite element and finite difference software packages.

Over the past two decades, the diffuse interface method (DIM) has gained widespread attention and is regarded as an effective alternative approach to sharp interface methods for solving PDE problems on complex geometries. This method represents the physical domain implicitly using a phase-field function, which can be interpreted as the domain’s indicator function when the interface thickness approaches zero and is often coupled with classic discretization schemes, such as finite difference method, finite element method [44], spectral method [12], and Nitsche’s method [36]. The DIM has been applied to elliptic interface problems, two-phase flow problems, and various material and engineering applications. Kockelkoren et al. [29] were the first to apply the diffuse interface method to study diffusion inside a cell with zero Neumann boundary conditions. Many numerical methods have been developed for solving different two-phase flow problems based on DIM. For example, Liu et al. combined the DIM with the consistent and conservative phase-field method to solve two-phase flows in complex geometries [35]. The DIM was applied to solve two-phase flows of viscous, incompressible fluids with matched densities, leading to coupled Navier-Stokes or Cahn-Hilliard systems [1, 23]. Moreover, the diffuse interface method can address miscible fluids of different densities [2], compressible fluids [20], Stokes-Darcy coupled equations [13], and problems involving more than two phases, where additional labeling functions are introduced to distinguish among them [11]. The diffuse interface method also has been extensively used to solve various models, such as the patient-specific human liver model based on MRI scans [40], the PDEs in moving geometries [19], and variational inverse problems [14]. Furthermore, in computational materials science, the diffuse interface method has been used to incorporate crystal plasticity into multiphase-field formulations by treating grain boundaries as finite-thickness diffuse interfaces [28], to develop high-order schemes for simulating multi-material systems undergoing large elastic–plastic deformations [42], and to design grain-boundary tracking strategies that embed crystal plasticity within the multiphase-field framework [37], among other applications.

The diffuse domain method (DDM) [6, 38], can be viewed as a variant of DIM. The DDM represents the original physical domain implicitly using a phase-field function with a narrow diffuse interface layer, wherein the value of the phase-field function rapidly transits from 1 inside the domain to 0 outside the domain [30]. The original PDE problem is then subsequently reformulated into a similar problem in a larger rectangular domain. As a result, the challenges associated with mesh generation for complex domains are mitigated, enabling the straightforward generation of spatial meshes for the rectangular domain to solve the transformed PDE problem with existing numerical schemes. The DDM has been used to solve PDEs in complex, stationary, or moving geometries with Dirichlet, Neumann, and Robin boundary conditions [33]. The properties and convergence of the diffuse domain method have been analyzed in several studies. Li et al. demonstrated that, in the diffuse domain method, several approximations to the physical boundary conditions converge asymptotically to the correct sharp interface problem [33]. They also observed that the choice of boundary condition can significantly affect numerical accuracy. Furthermore, Lervag et al. discussed that for specific choices of boundary condition approximations, the asymptotic convergence of the diffuse domain method can be improved to second order [30]. Lowengrub et al. have applied this method to elliptic problems and provided an asymptotic analysis of the boundary layer in [4, 30]. Additionally, Franz et al. analyzed the error estimates in the $L^{\infty }$-norm for one-dimensional elliptic equations [21]. Numerical errors in $L^2$, $L^{\infty }$, and $H^1$-norms on the original region have been further investigated for elliptic problems with Dirichlet boundary conditions [39]. Burger et al. constructed weighted Sobolev spaces based on the phase-field function and analyzed the approximation error in the extended region within the weighted $L^2$-norm [15]. Moreover, the convergence rate on the original domain for the Stokes-Darcy coupled problem has been discussed in [13]. Guo et al. coupled the DDM with an interface model to simulate two-phase fluid flows with variable physical properties while maintaining thermodynamic consistency [25]. The DDM approach was also employed to develop biomedical models, such as the chemotaxis-fluid diffuse-domain model for simulating bioconvection [41], a needle insertion model [27], etc.

In this paper, we are concerned with the diffuse domain method for solving the following linear second-order parabolic equation with a Neumann boundary condition:

1 $$\begin{aligned} \left\{ \begin{array}{ll} \, u_t = \nabla \cdot (A\nabla u) + f(t), \quad &  {\boldsymbol{x}} \in D,\quad 0 \le t \le T\\ \, u|_{t=0} = u_0, \quad &  {\boldsymbol{x}} \in D,\\ \, (A\nabla u) \cdot {\boldsymbol{n}} = g(t), \quad &  {\boldsymbol{x}} \in \partial D, \quad 0 \le t \le T, \end{array} \right. \end{aligned}$$

where *D* is an open bounded Lipschitz domain with irregular shape in $\mathbb {R}^d$ ($d \ge 1$), $T>0$ is the terminal time, $A({\boldsymbol{x}})>0$ is the diffusion coefficient and fulfills $\kappa \le A({\boldsymbol{x}})\le \kappa ^{-1}$ for all ${\boldsymbol{x}} \in D$ with some constant $\kappa >0$, $u(t,{\boldsymbol{x}})$ is the unknown function, $f(t,{\boldsymbol{x}})$ is the source term, $u_0({\boldsymbol{x}})$ is the initial value, and $g(t,{\boldsymbol{x}})$ is the Neumann boundary value. We will analyze the convergence of the diffuse domain solution as the interface thickness parameter goes to zero and derive corresponding error estimates measured in the weighted $L^2$ and weighted $H^1$ norms. The analysis techniques mainly follow the weighted Sobolev space-based framework developed in [15], but also with some significant enhancements. Our results successfully decouple the relationship between the hidden constants and the interface thickness parameter in the error estimates. Furthermore, the convergence rates are also improved to second order in the weighted $L^2$ norm and first order in the weighted $H^1$ norm, which are optimal as verified by the numerical experiments. To the best of our knowledge, this work presented in this paper is the first study on rigorous error analysis of the DDM for the second-order parabolic equations.

The rest of the paper is organized as follows. The diffuse domain method for the model problem (1) is first described in Section 2, and several preliminaries and lemmas for the weighted Sobolev spaces are given in Section 3. The convergence of the diffuse domain solution to the original solution as the interface thickness parameter goes to zero is proved in Section 4, together with the corresponding optimal error estimates under the weighted $L^2$ and $H^1$ norms. In Section 5, some numerical experiments are carried out to verify the theoretical results. Finally, some concluding remarks are drawn in Section 6.

## The diffuse domain method

First of all, some standard notations are proposed for later provement. For a given open bounded Lipschitz domain $\Omega \subset \mathbb {R}^d$ and for nonnegative integer *s*, denote $H^s(\Omega )$ as the standard integer-order Sobolev spaces on $\Omega$ with norm $\Vert \cdot \Vert _{s,\Omega }$ and semi-norm $|\cdot |_{s,\Omega }$, and the corresponding $L^2$-inner product is $(\cdot ,\cdot )_{\Omega }$. The corresponding norm of space $H^s(\Omega )$ is $\Vert \cdot \Vert _{s,\Omega }$ and $\Vert v\Vert _{k,\infty ,\Omega }=\textrm{ess}\sup _{|{\boldsymbol{\alpha }}|\le k}\Vert D^{{\boldsymbol{\alpha }}}v\Vert _{L^{\infty }(\Omega )}$ for any function *v* such that the right-hand side term makes sense, where ${\boldsymbol{\alpha }}=(\alpha _1, \cdots , \alpha _d)$ is a multi-index and $|{\boldsymbol{\alpha }}|=\alpha _1+\cdots +\alpha _d$. $H_0^s(\Omega )$ is the closure of $C_0^{\infty }(\Omega )$ with homogeneous Dirichlet boundary conditions. Moreover, given two quantities *a* and *b*, $a \lesssim b$ is the abbreviation of $a \le Cb$, where the hidden constant *C* is positive and independent of the mesh size; $a\eqsim b$ is equivalent to $a\lesssim b \lesssim a$.

Assume that $u_0\in H^2(D)$, $A\in H^1(D)$, $g(t)\in L^2(0,T;L^2(\partial D))$ and $f(t)\in L^2(0,T;L^2(D))$, then the variational formulation of (1) is given by: find $u \in L^2(0,T;H^1(D))$ and $u_t \in L^2(0,T;L^2(D))$ such that

2 $$\begin{aligned} \left\{ \begin{array}{ll} \,(u_t,v)+a(u,v)=\ell (v), \qquad &  \forall \,v \in H^1(D),\ 0 \le t \le T,\\ \,u(0,{\boldsymbol{x}})=u_0({\boldsymbol{x}}), \end{array} \right. \end{aligned}$$

where the bilinear operator $a(\cdot ,\cdot )$ is symmetric positive and defined by

3 $$\begin{aligned} a(w,v)=\int _{D} A\nabla w\cdot \nabla v \,\textrm{d}{\boldsymbol{x}}, \qquad \forall \,w, v\in H^1(D), \end{aligned}$$

and the linear operator $\ell (\cdot )$ is defined by

4 $$\begin{aligned} \ell (v)=\int _{D} fv\,\textrm{d}{\boldsymbol{x}} + \int _{\partial D} gv\,\textrm{d}\sigma ,\qquad \forall \, v\in H^1(D), \end{aligned}$$

Next, we apply the DDM to approximate the above integrals on the domain *D* [15]. First, let us introduce a signed distance function $d_D({\boldsymbol{x}})=\,\textrm{dist}({\boldsymbol{x}},D)-\,\textrm{dist}({\boldsymbol{x}},\mathbb {R}^n\setminus D)$, ${\boldsymbol{x}} \in \mathbb {R}^n$. It’s obvious that the domain *D* can be represented as $D=\{{\boldsymbol{x}}\,|\,d_D({\boldsymbol{x}})<0 \}$ with $\partial D=\{{\boldsymbol{x}}\,|\,d_D({\boldsymbol{x}})=0 \}$. To relax the sharp interface representation, let us further introduce $\varphi ^{\epsilon }({\boldsymbol{x}})=S(-d_D({\boldsymbol{x}})/\epsilon )$, where $\epsilon >0$ is a small interface thickness parameter and *S* being a smooth function such that $S(s) = -1$ for $s< -1$, $S(s) = 1$ for $s> 1$, and monotonic transition occurs when $s\in (-1,1)$. For instance, the sigmoidal function

5 $$\begin{aligned} S(s) = \tanh (3s) \end{aligned}$$

is often taken and we follow it in this paper. As $\epsilon$ tends to zero, $S(\cdot /\epsilon )$ converges to the sign function, and hence, the phase-field function

6 $$\begin{aligned} \omega _{\epsilon }({\boldsymbol{x}}):=(1+\varphi ^{\epsilon }({\boldsymbol{x}}))/2 \end{aligned}$$

converges to the indicator function $\chi _D$ of *D*. Define $D_s=\{{\boldsymbol{x}}\,|\,d_D({\boldsymbol{x}})<s \}$, $s \in (-\epsilon ,\epsilon )$. It is easy to find that $\omega _{\epsilon }=0$ for ${\boldsymbol{x}} \notin D_{\epsilon }$, $0\le \omega _{\epsilon }\le \frac{1}{2}$ for ${\boldsymbol{x}} \in D_{\epsilon }\setminus D$, $\frac{1}{2}\le \omega _{\epsilon }\le 1$ for ${\boldsymbol{x}} \in D\setminus D_{-\epsilon }$ and $\omega _{\epsilon }=1$ for ${\boldsymbol{x}} \in D_{-\epsilon }$. Furthermore, we can easily find that $|S'(s)|\lesssim 1$ for all *s*, and

7 $$\begin{aligned} \int _{-\epsilon }^{\epsilon } \frac{3}{2\epsilon }S'\left( -\frac{3s}{\epsilon }\right) \,\textrm{d}s=1.\end{aligned}$$

The key idea of DDM is to use a weighted averaging of the integrals over $D_s$, instead of integrating over the original irregular domain $D=D_0$ only. In order to generate the spatial mesh conveniently, one usually further fixes a larger rectangular domain $\Omega$ such that $D\subset D_{\epsilon } \subset \Omega$ in practice. Fig. 2 describes the geometric relation among the original physical domain *D*, the $\epsilon$-extension domain $D_{\epsilon }$ and the covering rectangular domain $\Omega$. Since $\omega _{\epsilon }=0$ for ${\boldsymbol{x}} \notin D_{\epsilon }$, we can compute the weighted averaging of the integrals over regular domain $\Omega$.

**Fig. 1:**
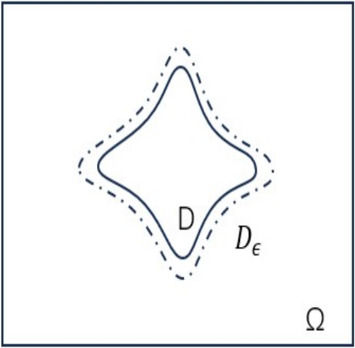
The relationship among *D*, $D_{\epsilon }$ and $\Omega$ for some $\epsilon >0$

Following the similar arguments of [15], consider the approximation of boundary integration over $\partial D$. Since $\frac{3}{2\epsilon } S'(\frac{3s}{\epsilon })$ approximates a concentrated distribution at zero, we expect for any integrable function $h:\Omega \rightarrow \mathbb {R}$,

$$\begin{aligned} \begin{aligned} \int _D h({\boldsymbol{x}})\,\textrm{d}{\boldsymbol{x}}&=\int _{-\infty }^{\infty } \frac{3}{2\epsilon } S'\left( -\frac{3s}{\epsilon }\right) \int _{D_0} h({\boldsymbol{x}})\,\textrm{d}{\boldsymbol{x}}\,\textrm{d}s\\&\approx \int _{-\epsilon }^{\epsilon } \frac{3}{2\epsilon } S'\left( -\frac{3s}{\epsilon }\right) \int _{D_s} h({\boldsymbol{x}})\,\textrm{d}{\boldsymbol{x}} \,\textrm{d}s=\frac{1}{2}\int _{-1}^{1}\int _{\{\varphi ^{\epsilon }>s\}} h({\boldsymbol{x}})\,\textrm{d}{\boldsymbol{x}} \,\textrm{d}s. \end{aligned} \end{aligned}$$

Using the Fubini’s theorem, we may further rewrite

8 $$\begin{aligned} \int _{-1}^1 \int _{\{\varphi ^{\epsilon }>s \}}h({\boldsymbol{x}})\,\textrm{d}{\boldsymbol{x}}\,\textrm{d}s = \int _{D_{\epsilon }}\int _{-1}^{\varphi ^{\epsilon }({\boldsymbol{x}})}\,\textrm{d}s\ h({\boldsymbol{x}})\,\textrm{d}{\boldsymbol{x}} = \int _{D_{\epsilon }}(1+\varphi ^{\epsilon }({\boldsymbol{x}}))h({\boldsymbol{x}})\,\textrm{d}{\boldsymbol{x}}. \end{aligned}$$

By the co-area formula, we then derive an approximation for the boundary integral as

9 $$\begin{aligned} \begin{aligned} \int _{\partial D} h({\boldsymbol{x}})\,\textrm{d}\sigma ({\boldsymbol{x}}) \approx&\; \frac{1}{2}\int _{-1}^1\int _{\partial \{\varphi ^{\epsilon }>s \}}h({\boldsymbol{x}})\,\textrm{d}\sigma ({\boldsymbol{x}})\,\textrm{d}s\\ =&\;\frac{1}{2}\int _{D_{\epsilon }} h({\boldsymbol{x}})\left| \nabla \varphi ^{\epsilon }({\boldsymbol{x}})\right| \,\textrm{d}{\boldsymbol{x}}\\ =&\int _{D_{\epsilon }} h({\boldsymbol{x}})\left| \nabla \omega ^{\epsilon }({\boldsymbol{x}})\right| \,\textrm{d}{\boldsymbol{x}}. \end{aligned} \end{aligned}$$

Let us define the weighted $L^p$ space on $D_{\epsilon }$, $1\le p < \infty$, associated with the phase-field function $\omega _{\epsilon }$ as follows:

$$\begin{aligned} L^p(D_{\epsilon };\omega _{\epsilon })=\left\{ v\,\Big |\,\int _{D_{\epsilon }} |v|^p\,\omega _{\epsilon }\,\textrm{d}{\boldsymbol{x}}<\infty \right\} , \end{aligned}$$

with the norm

$$\begin{aligned} \Vert v\Vert _{L^p(D_{\epsilon };\omega _{\epsilon })}=\left( \int _{D_{\epsilon }}|v|^p \,\omega _{\epsilon }\,\textrm{d}\omega _{\epsilon }\right) ^{\frac{1}{p}}. \end{aligned}$$

Based on the weighted spaces $L^p(D_{\epsilon };\omega _{\epsilon })$, the weighted Sobolev spaces are consequently defined as

$$\begin{aligned} W^{s,p}(D_{\epsilon };\omega _{\epsilon })=\left\{ v\in L^p(D_{\epsilon };\omega _{\epsilon })\,|\, D^{{\boldsymbol{\alpha }}}v \in L^p(D_{\epsilon };\omega _{\epsilon }), \ \forall \, |{\boldsymbol{\alpha }}|\le s \right\} , \end{aligned}$$

and $H^s(D_{\epsilon };\omega ^{\epsilon }):=W^{s,2}(D_{\epsilon };\omega _{\epsilon })$ with the norm

$$\begin{aligned} \Vert v\Vert _{W^{s,p}(D_{\epsilon };\omega _{\epsilon })}=\left( \int _{D_{\epsilon }}\sum \limits _{|{\boldsymbol{\alpha }}|\le s}\left| D^{{\boldsymbol{\alpha }}}v\right| ^p\,\textrm{d}\omega _{\epsilon }\right) ^{\frac{1}{p}}. \end{aligned}$$

Therefore, (8) and (9) leads to the following diffuse domain approximation of (2) in $D_{\epsilon }$: find $u^{\epsilon } \in L^2(0,T;H^1(D_{\epsilon };\omega _{\epsilon }))$ such that

10 $$\begin{aligned} \left( u_t^{\epsilon },v \right) _{D_{\epsilon };\omega _{\epsilon }}+a^{\epsilon }(u^{\epsilon },v)= \ell ^{\epsilon }(v), \qquad \forall \,v \in H^1(D_{\epsilon };\omega _{\epsilon }), \end{aligned}$$

where

$$\begin{aligned} \begin{aligned} a^{\epsilon }(w,v)&= \int _{D_{\epsilon }} \widetilde{A}\nabla w\cdot \nabla v \,\omega _{\epsilon }\,\textrm{d}{\boldsymbol{x}},\qquad \forall \,w, v\in H^1(D_{\epsilon };\omega _{\epsilon }),\\ \ell ^{\epsilon }(v)&= \int _{D_{\epsilon }} \widetilde{f} v\omega _{\epsilon }\,\textrm{d}{\boldsymbol{x}} + \int _{D_{\epsilon }} \widetilde{g}v\left| \nabla \omega _{\epsilon } \right| \,\textrm{d}{\boldsymbol{x}},\qquad \forall \, v\in H^1(D_{\epsilon };\omega _{\epsilon }), \end{aligned} \end{aligned}$$

with the initial value $u^{\epsilon }(0) = \widetilde{u_0}$. Here $\widetilde{A}$, $\widetilde{u_0}$, $\widetilde{f}(t)$ and $\widetilde{g}(t)$ are certain extensions of *A*, $u_0$, *f*(*t*) and *g*(*t*) from *D* to $D_{\epsilon }$, respectively. The extension details will be illustrated in the following.

Let us also assume the domain boundary $\partial D$ is of class $C^{2,1}$ from now on in this paper. Define the $\epsilon$-tubular neighborhood of $\partial D$ by

$$\begin{aligned} \Gamma _{\epsilon } = D_{\epsilon }\setminus \overline{D_{-\epsilon }}. \end{aligned}$$

It holds that $\,\textrm{dist}({\boldsymbol{x}},\partial D)< \epsilon$ for all ${\boldsymbol{x}} \in \Gamma _{\epsilon }$ and $\,\textrm{dist}({\boldsymbol{x}},\partial D)\ge \epsilon$ for all ${\boldsymbol{x}} \in \Omega \setminus \Gamma _{\epsilon }$. Due to the $C^{2,1}$ regularity of $\partial D$, the projection of ${\boldsymbol{z}} \in \Gamma _{\epsilon }$ onto $\partial D$ is unique for sufficiently small $\epsilon$ [3], i.e., for each $z \in \Gamma _{\epsilon }$, there exists a unique ${\boldsymbol{x}} \in \partial D$ such that ${\boldsymbol{z}} = {\boldsymbol{x}}+d_{D}({\boldsymbol{z}}){\boldsymbol{n}}({\boldsymbol{x}})$, where ${\boldsymbol{n}}({\boldsymbol{x}})$ is the outward unit normal vector for ${\boldsymbol{x}}\in \partial D$. To preserve the regularity of the source function *f*(*t*), the diffusion coefficient *A* and the initial value $u_0$, one can, for example, extend them by the reflection with respect to the outward normal direction of the domain boundary $\partial D$. For $L^p$-functions, this can be achieved by an extension by constant and for the $W^{k,p}$-functions, this can be achieved by the construction shown in the Chapter 4 of [3]. Regarding the extension of Neumann boundary value, since $g(t) \in L^2(\partial D)$, *g*(*t*) is defined a.e. on $\partial D$ and we can define an extension of *g*(*t*) a.e. on $\Gamma _{\epsilon }$ by

$$\begin{aligned} \widetilde{g}(t,{\boldsymbol{x}}+s{\boldsymbol{n}}({\boldsymbol{x}}))=g(t,{\boldsymbol{x}}), \quad -\epsilon \le s\le \epsilon , \ {\boldsymbol{x}} \in \partial D, \end{aligned}$$

and further simply take $\widetilde{g}(t,{\boldsymbol{x}})=0$ for ${\boldsymbol{x}}\in D_{\epsilon }\setminus \Gamma _{\epsilon }$.

Based on the definition of weight function $\omega _{\epsilon }$, it’s obvious that $|\nabla \omega _{\epsilon }({\boldsymbol{x}})|\ne 0$ only for ${\boldsymbol{x}} \in \Gamma _{\epsilon }$. Since the weighted function $\omega _{\epsilon }({\boldsymbol{x}})$ and its gradient $\nabla \omega _{\epsilon }({\boldsymbol{x}})$ will vanish on $\partial D_{\epsilon }$ and outside of $D_{\epsilon }$, we can further extend the integration on $D_{\epsilon }$ to the larger rectangular domain $\Omega$, which is easy for generating spatial grids in practice. The weighted Sobolev space and corresponding variational problem (2) defined in $D_{\epsilon }$ is then transformed to a similar problem defined in $\Omega$: find $u^{\Omega } \in L^2(0,T;H^1(\Omega ;\omega _{\epsilon }))$ such that

11 $$\begin{aligned} \left( u_t^{\Omega },v\right) _{\Omega ;\omega _{\epsilon }}+a^{\Omega }(u^{\Omega },v)= \ell ^{\Omega }(v), \quad \forall \, v \in H^1(\Omega ;\omega _{\epsilon }), \end{aligned}$$

where

$$\begin{aligned} \begin{aligned} a^{\Omega }(w,v)&= \int _{\Omega } \overline{A}\nabla w\cdot \nabla v \,\omega _{\epsilon }\,\textrm{d}{\boldsymbol{x}},\qquad \forall \,w, v\in H^1(\Omega ;\omega _{\epsilon }),\\ \ell ^{\Omega }(v)&= \int _{\Omega } \overline{f}v\,\omega _{\epsilon }\,\textrm{d}{\boldsymbol{x}} + \int _{\Omega } \overline{g}v\left| \nabla \omega _{\epsilon } \right| \,\textrm{d}{\boldsymbol{x}},\qquad \forall v\in H^1(\Omega ;\omega _{\epsilon }) \end{aligned} \end{aligned}$$

with the initial value $u^{\Omega }(0)=\overline{u_0}$. Here $\overline{A}$, $\overline{u_0}$, $\overline{f}(t)$ and $\overline{g}(t)$ are the extensions of $\widetilde{A}$, $\widetilde{u_0}({\boldsymbol{x}})$, $\widetilde{f}(t)$ and $\widetilde{g}(t)$ from $D_{\epsilon }$ to $\Omega$ and the choices of extensions are not unique. Since $\omega _{\epsilon }({\boldsymbol{x}})=0$ for ${\boldsymbol{x}} \in \Omega \setminus D_{\epsilon }$ and consequently $u^{\epsilon }|_{D_{\epsilon }}=u^{\Omega }|_{D_{\epsilon }}$, we only need investigate convergence of the diffuse domain solution $u^\epsilon$ defined on $D_{\epsilon }$ produced from the variational problem (10) to the exact solution *u* of the original variational problem (2).

## Preliminaries

Note that $\Gamma _{\epsilon }$ can be rewritten as

$$\begin{aligned} \Gamma _{\epsilon }=\left\{ {\boldsymbol{z}} \in \Omega \,\big |\, \exists \ {\boldsymbol{x}} \in \partial D,\ |s|<\epsilon ,\ {\boldsymbol{z}} ={\boldsymbol{x}} + s{\boldsymbol{n}}({\boldsymbol{x}}) \right\} . \end{aligned}$$

Furthermore, $\left| \Gamma _{\epsilon } \right| \lesssim \epsilon \mathcal {H}^{n-1}(\partial D)$, where $\left| \Gamma _{\epsilon }\right| =\mathcal {L}^n(\Gamma _{\epsilon })$ is the *n*-dimensional Lebesgue measure of $\Gamma _{\epsilon }$ and $\mathcal {H}^{n-1}(\partial D)$ is the $(n-1)$-dimensional Hausdorff measure of $\partial D$.

### On the weighted Sobolev space

The following three theorems (Theorems 1, 2 and 3) have readily been proved in [15].

#### Theorem 1

(Trace Theorem) Let $\epsilon _0>0$ be sufficiently small and $1\le p < \infty$. Then, there exists a constant $C>0$ such that for any $\epsilon \in [0, \epsilon _0]$ and $v \in W^{1,p}(D_{\epsilon };\omega _{\epsilon })$, there holds

$$\begin{aligned} \int _{D_{\epsilon }} |v|^p|\nabla \omega _{\epsilon }|\,\textrm{d}{\boldsymbol{x}} \lesssim \Vert v\Vert _{W^{1,p}(D_{\epsilon };\omega _{\epsilon })}^p. \end{aligned}$$

#### Theorem 2

(Embedding Theorem) Suppose that $\epsilon \in (0, \epsilon _0]$, $1\le p < \infty$, and $\alpha >0$ be the constant satisfies that for all $s \in (0, 2)$, $\zeta _1 s^{\alpha }\le (1+S(s-1))/2\le \zeta _2 s^{\alpha }$ for some constants $\zeta _1,\zeta _2>0$. Then the following embeddings are continuous

$$\begin{aligned} W^{1,p}(D_{\epsilon };\omega _{\epsilon }) \hookrightarrow L^q(D_{\epsilon };\omega _{\epsilon }), \quad 1 \le q \le p_{\alpha }^*, \ q < \infty , \end{aligned}$$

where $p_{\alpha }^*=\frac{p(n+\alpha )}{n+\alpha -p}$ for $p < n+\alpha$ and $p_{\alpha }^*=\infty$ for $p \ge n+\alpha .$ Moreover, there exists a constant *C* independent of $\epsilon$ such that for any $v \in W^{1,p}(D_{\epsilon };\omega _{\epsilon })$, there holds

$$\begin{aligned} \Vert v\Vert _{L^q}(D_{\epsilon };\omega _{\epsilon }) \lesssim \Vert v\Vert _{W^{1,p}(D_{\epsilon };\omega _{\epsilon })}. \end{aligned}$$

#### Theorem 3

(Poincare-Friedrichs-type inequality) Suppose that $\epsilon \in (0,\epsilon _0]$, $1\le p<\infty$, and the extended domain $D_{\epsilon }$ be connected. Then, there exists a constant *C* independent of $\epsilon$ such that for any $\epsilon \in (0,\epsilon _0)$ and $v \in W^{1,p}(D_{\epsilon };\omega _{\epsilon })$, there holds

$$\begin{aligned} \Vert v\Vert _{L^p(D_{\epsilon };\omega _{\epsilon })} \lesssim \Vert \nabla v\Vert _{L^p(D_{\epsilon };\omega _{\epsilon })}^p + \int _{D_{\epsilon }}|v|^p|\nabla \omega _{\epsilon }|\,\textrm{d}{\boldsymbol{x}}. \end{aligned}$$

### On the diffuse volume integrals

The following two theorems readily come from Theorem 5.2 and Theorem 5.6 in [15].

#### Theorem 4

Suppose that $\epsilon \in (0, \epsilon _0]$ and $h({\boldsymbol{x}}) \in H^1(D_{\epsilon };\omega _{\epsilon })$. Then,

$$\begin{aligned} \left| \int _{D_{\epsilon }} h({\boldsymbol{x}})\,\textrm{d}\omega _{\epsilon }({\boldsymbol{x}})-\int _D h({\boldsymbol{x}})\,\textrm{d}{\boldsymbol{x}} \right| \lesssim \epsilon ^{\frac{3}{2}}\Vert h\Vert _{H^1(\Gamma _{\epsilon };\omega _{\epsilon })}, \end{aligned}$$

where the hidden constant is independent of $\epsilon$.

#### Theorem 5

Suppose that $\epsilon \in (0, \epsilon _0]$, $w \in H^2(D_{\epsilon };\omega _{\epsilon })$ satisfying $w=0$ on $\partial D$ and $v \in H^1(D_{\epsilon };\omega _{\epsilon })$. Then, there holds

$$\begin{aligned} \int _{\Gamma _{\epsilon }} wv\left| \nabla \omega _{\epsilon }\right| \,\textrm{d}{\boldsymbol{x}} \lesssim \left( \epsilon ^{\frac{3}{2}}\Vert w\Vert _{H^2(D_{\epsilon };\omega _{\epsilon })}+\epsilon ^{\frac{3}{2}}\Vert w\Vert _{H^2(\Gamma _{\epsilon };\omega _{\epsilon })}\right) \Vert v\Vert _{H^1(D_{\epsilon };\omega _{\epsilon })}, \end{aligned}$$

where the hidden constant is independent of $\epsilon$, *w* and *v*.

Next, let us introduce the smooth condition for *f* and some regularity condition required for the exact solution *u* in order to carry out the convergence and error analysis of the diffuse domain method. For the simplicity of expressions, in the following analysis we will not distinguish the exact solution *u*(*t*), the source function *f*(*t*), the Neumann boundary value *g*(*t*), the diffusion coefficient *A* with their extensions in $D_\epsilon$ since there are no ambiguities.

#### Assumption 1

The extension of the source function $f(t,{\boldsymbol{x}})$ to $D_{\epsilon }$ satisfies the following regularity condition:

12a $$\begin{aligned} \sup \limits _{0\le t \le T}\Vert f(t,{\boldsymbol{x}})\Vert _{L^2(D_{\epsilon };\omega _{\epsilon })}&\lesssim 1,\end{aligned}$$

12b $$\begin{aligned} \sup \limits _{0\le t \le T}\Vert f(t,{\boldsymbol{x}})\Vert _{H^1(\Gamma _{\epsilon };\omega _{\epsilon })}&\lesssim 1, \end{aligned}$$

where the hidden constants may depend on the terminal time *T*.

#### Assumption 2

The extension of the exact solution *u*(*t*) of (2) to $D_\epsilon$ satisfies the following regularity conditions:

13a $$\begin{aligned} \sup \limits _{0\le t \le T}\Vert u(t)\Vert _{H^2(D_{\epsilon };\omega _{\epsilon })}&\lesssim 1,\end{aligned}$$

13b $$\begin{aligned} \sup \limits _{0\le t \le T}\Vert u_t(t)\Vert _{W^{1,\infty }(D_{\epsilon })}&\lesssim 1, \end{aligned}$$

where the hidden constants may depend on the terminal time *T*.

In the following part, in order to obtain sharp error estimates for the DDM, we will fully use the property $|\Gamma _{\epsilon }|\lesssim \epsilon$ and the regularity of *u*, which lead to optimal error estimates with respect to the weighted $L^2$-norm and the weighted $H^1$-norm.

#### Lemma 1

Suppose that $0<\epsilon \le \epsilon _0$, $w\in H^1(D_{\epsilon };\omega _{\epsilon })\cap L^{\infty }(D_{\epsilon })$, and $v\in H^1(D_{\epsilon };\omega _{\epsilon })$. Then

$$\begin{aligned} \left| \int _{D_{\epsilon }}w v\omega _{\epsilon }\,\textrm{d}{\boldsymbol{x}}-\int _D w v\,\textrm{d}{\boldsymbol{x}} \right| \lesssim \epsilon ^{\frac{1}{2}} \Vert w\Vert _{L^2(\Gamma _{\epsilon };\omega _{\epsilon })}\Vert v\Vert _{L^2(\Gamma _{\epsilon };\omega _{\epsilon })}, \end{aligned}$$

where the hidden constant is independent of $\epsilon$, and linearly dependent of *D*.

#### Proof

According to the definition of $D_{\epsilon }$ and $\omega _{\epsilon }$, we can rewrite

14 $$\begin{aligned} \begin{aligned}&\int _{D_{\epsilon }}w v\omega _{\epsilon } \,\textrm{d}{\boldsymbol{x}}-\int _D w v\,\textrm{d}{\boldsymbol{x}}\\&\quad =\int _{D_{-\epsilon }}wv\omega _{\epsilon } \,\textrm{d}{\boldsymbol{x}}+\int _{D_{\epsilon }\setminus D_{-\epsilon }}wv\omega _{\epsilon }\,\textrm{d}{\boldsymbol{x}}-\int _{D_{-\epsilon }}wv\,\textrm{d}{\boldsymbol{x}}-\int _{D\setminus D_{-\epsilon }} wv\,\textrm{d}{\boldsymbol{x}}\\&\quad =\int _{D\setminus D_{-\epsilon }}wv\omega _{\epsilon }\,\textrm{d}{\boldsymbol{x}}+\int _{D_{\epsilon }\setminus D}wv\omega _{\epsilon }\,\textrm{d}{\boldsymbol{x}}-\int _{D\setminus D_{-\epsilon }} wv\,\textrm{d}{\boldsymbol{x}}\\&\quad =\int _{D\setminus D_{-\epsilon }} \left( w-w/\omega _{\epsilon }\right) v\omega _{\epsilon }\,\textrm{d}{\boldsymbol{x}}+\int _{D_{\epsilon }\setminus D}wv\omega _{\epsilon }\,\textrm{d}{\boldsymbol{x}}. \end{aligned} \end{aligned}$$

By applying the Cauchy-Schwarz and triangle inequalities, (14) can be bounded as

15 $$\begin{aligned} \begin{aligned}&\int _{D_{\epsilon }}w v\omega _{\epsilon } \,\textrm{d}{\boldsymbol{x}}-\int _D w v\,\textrm{d}{\boldsymbol{x}}\\&\quad \le \left( \int _{D\setminus D_{-\epsilon }}\left| w-w/\omega _{\epsilon } \right| ^2\omega _{\epsilon }\,\textrm{d}{\boldsymbol{x}} \right) ^{\frac{1}{2}}\left( \int _{D\setminus D_{-\epsilon }}|v|^2\omega _{\epsilon }\,\textrm{d}{\boldsymbol{x}}\right) ^{\frac{1}{2}}\\&\quad \quad +\left( \int _{D_{\epsilon }\setminus D}|w|^2\omega _{\epsilon }\,\textrm{d}{\boldsymbol{x}}\right) ^{\frac{1}{2}}\left( \int _{D_{\epsilon }\setminus D} |v|^2\omega _{\epsilon }\,\textrm{d}{\boldsymbol{x}}\right) ^{\frac{1}{2}}\\&\quad \lesssim \left( \int _{\Gamma _{\epsilon }} |v|^2\omega _{\epsilon } \,\textrm{d}{\boldsymbol{x}}\right) ^{\frac{1}{2}}\left( \int _{D\setminus D_{-\epsilon }}\left| w-w/\omega _{\epsilon } \right| ^2\omega _{\epsilon }\,\textrm{d}{\boldsymbol{x}}+\int _{D_{\epsilon }\setminus D} |w|^2\omega _{\epsilon }\,\textrm{d}{\boldsymbol{x}} \right) ^{\frac{1}{2}}\\&\quad \lesssim \left( \int _{\Gamma _{\epsilon }} |v|^2\omega _{\epsilon } \,\textrm{d}{\boldsymbol{x}}\right) ^{\frac{1}{2}} \Big (\mathrm{{I}}_1+\mathrm{{I}}_2 \Big )^{\frac{1}{2}} \end{aligned} \end{aligned}$$

where

$$\begin{aligned} \mathrm{{I}}_1:=\int _{D_{\epsilon }\setminus D_{-\epsilon }} |w|^2\omega _{\epsilon }\,\textrm{d}{\boldsymbol{x}} - \int _{D\setminus D_{-\epsilon }}|w|^2\,\textrm{d}{\boldsymbol{x}},\quad \mathrm{{I}}_2:=\int _{D\setminus D_{-\epsilon }}|w|^2/\omega _{\epsilon }\,\textrm{d}{\boldsymbol{x}}-\int _{D\setminus D_{-\epsilon }}|w|^2\,\textrm{d}{\boldsymbol{x}}. \end{aligned}$$

As for the estimate of $\textrm{I}_1$, with the help of Theorem 4, we can derive that

16 $$\begin{aligned} |\mathrm{{I}_1}|&= \left| \int _{D_{\epsilon }}|w|^2\omega _{\epsilon }\,\textrm{d}{\boldsymbol{x}}-\int _D|w|^2\,\textrm{d}{\boldsymbol{x}}\right| \lesssim \epsilon ^{\frac{3}{2}}\Vert w^2\Vert _{H^1(\Gamma _{\epsilon };\omega _{\epsilon })}. \end{aligned}$$

As for the estimate of $\textrm{I}_2$, recalling the definition of $D_{\epsilon }$ and $\omega _{\epsilon }$, we can derive that

17 $$\begin{aligned} \begin{aligned} |\mathrm{{I}_2}|=&\left| \int _{-\epsilon }^0 \frac{\frac{6}{\epsilon }S'\left( -\frac{3s}{\epsilon }\right) }{\left( 1+S\left( -\frac{3s}{\epsilon }\right) \right) ^2}\int _{\{d_D({\boldsymbol{x}})<s\}}|w|^2\,\textrm{d}{\boldsymbol{x}}\,\textrm{d}s -\int _{-\epsilon }^0\frac{\frac{6}{\epsilon }S'\left( -\frac{3s}{\epsilon }\right) }{\left( 1+S\left( -\frac{3s}{\epsilon }\right) \right) ^2}\int _{\{d_D({\boldsymbol{x}})<0\}}|w|^2\,\textrm{d}{\boldsymbol{x}}\,\textrm{d}s \right| \\ =&\left| \int _{-\epsilon }^0 \frac{\frac{6}{\epsilon }S'\left( -\frac{3s}{\epsilon }\right) }{\left( 1+S\left( -\frac{3s}{\epsilon }\right) \right) ^2}\int _{\{s<d_D({\boldsymbol{x}})<0\}}|w|^2\,\textrm{d}{\boldsymbol{x}}\,\textrm{d}s\right| \\ \lesssim&\, \frac{1}{\epsilon }\left( \int _{-\epsilon }^0 S'\left( -\frac{3s}{\epsilon }\right) \int _{\{s<d_D({\boldsymbol{x}})<0\}}1\,\textrm{d}{\boldsymbol{x}} \,\textrm{d}s\right) ^{\frac{1}{2}}\left( \int _{-\epsilon }^0 S'\left( -\frac{3s}{\epsilon }\right) \int _{\{s<d_D({\boldsymbol{x}})<0\}}|w|^4\,\textrm{d}{\boldsymbol{x}}\,\textrm{d}s \right) ^{\frac{1}{2}}\\ \lesssim&\, \epsilon ^{\frac{1}{2}}\left( \int _{-\epsilon }^0 \frac{1}{\epsilon } S'\left( -\frac{3s}{\epsilon }\right) \int _{\{s<d_D({\boldsymbol{x}})<0 \}}|w|^4\,\textrm{d}{\boldsymbol{x}}\,\textrm{d}s \right) ^{\frac{1}{2}}\\ \lesssim&\, \epsilon ^{\frac{1}{2}}\left( \int _0^1\int _{\{-s<\varphi ^{\epsilon }<0 \}}|w|^4\,\textrm{d}{\boldsymbol{x}}\,\textrm{d}s \right) ^{\frac{1}{2}}, \end{aligned} \end{aligned}$$

where the last three inequalities use the Cauchy-Schwarz inequality and the boundness of $S'(\cdot )$. Since $w \in L^{\infty }(D_{\epsilon })$, (17) can be reformulated as

18 $$\begin{aligned} \mathrm{{I}_2}&\lesssim \epsilon ^{\frac{1}{2}}\Vert w\Vert _{L^2(\Gamma _{\epsilon };\omega _{\epsilon })}. \end{aligned}$$

Combining (15), (16), (18) together, we finally obtain

$$\begin{aligned} \left| \int _{D_{\epsilon }} wv\omega _{\epsilon }\,\textrm{d}{\boldsymbol{x}}-\int _D wv\,\textrm{d}{\boldsymbol{x}} \right| \lesssim \epsilon ^{\frac{1}{2}}\Vert w\Vert _{L^2(\Gamma _{\epsilon };\omega _{\epsilon })}\Vert v\Vert _{L^2(\Gamma _{\epsilon };\omega _{\epsilon })}, \end{aligned}$$

which completes the proof. $\square$

## Error analysis

To illustrate the convergence of diffuse domain method, let us combine (2) and (10) together, then we can get the error equation as follows: for any $v \in H^1(D_{\epsilon };\omega _{\epsilon })$,

19 $$\begin{aligned} \begin{aligned} \left( u_t^{\epsilon }-u_t,v\right) _{D_{\epsilon };\omega _{\epsilon }}+a^{\epsilon }(u^{\epsilon }-u,v) =&[\left( u_t,v\right) -\left( u_t,v\right) _{D_{\epsilon };\omega _{\epsilon }}]+[a(u,v)-a^{\epsilon }(u,v)]\\&+[\ell ^{\epsilon }(v)-\ell (v)]. \end{aligned} \end{aligned}$$

Next we will derive the error estimates of $u^{\epsilon }-u$ in the weighted $L^2$ and $H^1$ norms.

### Optimal error estimate in the $L^2$-norm

#### Theorem 6

(Error estimate in the $L^2$ norm) Suppose that $0<\epsilon \le \epsilon _0$, $g(t)\in H^2(D_{\epsilon };\omega _{\epsilon })$ for $t\in (0,T]$, $\kappa \le A({\boldsymbol{x}})\le \kappa ^{-1}$ for all ${\boldsymbol{x}} \in D_{\epsilon }$ with some constant $\kappa >0$, and *f* satisfies Assumption 1. Assume the exact solution $u\in L^2(0,T;H^2(D))$ and its extension to $D_\epsilon$ fulfills Assumption 2. Then we have

20 $$\begin{aligned} \begin{aligned} \Vert u^{\epsilon }(t)-u(t)\Vert _{L^2(D_{\epsilon };\omega _{\epsilon })}\lesssim \epsilon ^2, \quad \forall \,0\le t \le T, \end{aligned} \end{aligned}$$

where the hidden constant is independent of $\epsilon$, and linearly dependent of *D*.

#### Proof

Let us analyze $\left( u_t,v\right) -\left( u_t,v\right) _{D_{\epsilon };\omega _{\epsilon }}$, $a(u,v)-a^{\epsilon }(u,v)$ and $\ell ^{\epsilon }(v)-\ell (v)$ respectively for any $v \in H^1(D_{\epsilon };\omega _{\epsilon })$. Recalling the definition of $a^{\epsilon }(\cdot ,\cdot )$ and $a(\cdot ,\cdot )$, since $\omega _{\epsilon }$ will vanish on $\partial D_{\epsilon }$, we can derive the following formula by using the integration by part,

21 $$\begin{aligned} \begin{aligned}&a(u,v)-a^{\epsilon }(u,v)\\&\quad =\int _{D_{\epsilon }} \,\textrm{div}(A\nabla u)\omega _{\epsilon }v\,\textrm{d}{\boldsymbol{x}}+\int _{D_{\epsilon }} A\nabla u\cdot \nabla \omega _{\epsilon } v\,\textrm{d}{\boldsymbol{x}}+\int _{\partial D} {\boldsymbol{n}}\cdot \left( A\nabla u\right) v\,\textrm{d}\sigma -\int _D\,\textrm{div}(A\nabla u)v\,\textrm{d}{\boldsymbol{x}}\\&\quad =\int _{D_{\epsilon }}\,\textrm{div}\left( A\nabla u\right) \omega _{\epsilon } v\,\textrm{d}{\boldsymbol{x}}-\int _D\,\textrm{div}\left( A\nabla u\right) v\,\textrm{d}{\boldsymbol{x}}-\int _{D_{\epsilon }} {\boldsymbol{n}}\cdot (A\nabla u)\left| \nabla \omega _{\epsilon }\right| v\,\textrm{d}{\boldsymbol{x}}+\int _{\partial D} gv\,\textrm{d}\sigma , \end{aligned} \end{aligned}$$

where in the last equality we use the fact that $\nabla \omega _{\epsilon }=-{\boldsymbol{n}}\left| \nabla \omega _{\epsilon }\right|$. By Theorem 4, we get

22 $$\begin{aligned}&\left| \int _D \,\textrm{div}(A\nabla u)v\,\textrm{d}{\boldsymbol{x}}-\int _{D_{\epsilon }} \,\textrm{div}(A\nabla u)v\omega _{\epsilon }\,\textrm{d}{\boldsymbol{x}}\right| \lesssim \epsilon ^{\frac{3}{2}}\left\| \,\textrm{div}(A\nabla u)\right\| _{H^1(\Gamma _{\epsilon };\omega _{\epsilon })}\Vert v\Vert _{H^1(\Gamma _{\epsilon };\omega _{\epsilon })}. \end{aligned}$$

Then, inserting (22) to (21), we get that

23 $$\begin{aligned} \begin{aligned} a(u,v)-a^{\epsilon }(u,v)\lesssim&\epsilon ^{\frac{3}{2}}\left\| \,\textrm{div}(A\nabla u)\right\| _{H^1(\Gamma _{\epsilon };\omega _{\epsilon })}\Vert v\Vert _{H^1(\Gamma _{\epsilon };\omega _{\epsilon })}-\int _{D_{\epsilon }} {\boldsymbol{n}}\cdot (A\nabla u)\left| \nabla \omega _{\epsilon }\right| v\,\textrm{d}{\boldsymbol{x}}\\&+\int _{\partial D} gv\,\textrm{d}\sigma . \end{aligned} \end{aligned}$$

As for the estimate of $\left( u_t,v\right) -\left( u_t,v\right) _{D_{\epsilon };\omega _{\epsilon }}$, by using the conclusion of Theorem 4, we obtain

24 $$\begin{aligned} \left( u_t,v\right) -\left( u_t,v\right) _{D_{\epsilon };\omega _{\epsilon }}\lesssim \epsilon ^{\frac{3}{2}}\Vert u_t\Vert _{H^1(\Gamma _{\epsilon };\omega _{\epsilon })}\Vert v\Vert _{H^1(\Gamma _{\epsilon };\omega _{\epsilon })}. \end{aligned}$$

As for the estimate of $\ell ^{\epsilon }(v)-\ell (v)$, same as the derivation of (24), we can get

25 $$\begin{aligned} \begin{aligned} \ell ^{\epsilon }(v)-\ell (v)&= \int _{D_{\epsilon }}fv\omega _{\epsilon }\,\textrm{d}{\boldsymbol{x}}-\int _D fv\,\textrm{d}{\boldsymbol{x}}+\int _{D_{\epsilon }}gv\left| \nabla \omega _{\epsilon }\right| \,\textrm{d}{\boldsymbol{x}}-\int _{\partial D}gv\,\textrm{d}\sigma \\&\lesssim \epsilon ^{\frac{3}{2}}\Vert f\Vert _{H^1(\Gamma _{\epsilon };\omega _{\epsilon })}\Vert v\Vert _{H^1(\Gamma _{\epsilon };\omega _{\epsilon })}+\int _{D_{\epsilon }} gv\left| \nabla \omega _{\epsilon }\right| \,\textrm{d}{\boldsymbol{x}}-\int _{\partial D}gv\,\textrm{d}\sigma . \end{aligned} \end{aligned}$$

Combining (23)-(25) together, we get

26 $$\begin{aligned} \begin{aligned}&\left( u_t^{\epsilon }-u_t,v\right) _{D_{\epsilon };\omega _{\epsilon }}+a^{\epsilon }(u^{\epsilon }-u,v)\\&\quad =\left( u_t,v\right) -\left( u_t,v\right) _{D_{\epsilon };\omega _{\epsilon }}+a(u,v)-a^{\epsilon }(u,v)+\ell ^{\epsilon }(v)-\ell (v)\\&\quad \lesssim \epsilon ^{\frac{3}{2}}\Vert u_t\Vert _{H^1(\Gamma _{\epsilon };\omega _{\epsilon })}\Vert v\Vert _{H^1(\Gamma _{\epsilon };\omega _{\epsilon })}+\epsilon ^{\frac{3}{2}}\left\| \,\textrm{div}\left( A\nabla u\right) \right\| _{H^1(\Gamma _{\epsilon };\omega _{\epsilon })}\Vert v\Vert _{H^1(\Gamma _{\epsilon };\omega _{\epsilon })}\\&\qquad -\int _{D_{\epsilon }} {\boldsymbol{n}}\cdot (A\nabla u)\left| \nabla \omega _{\epsilon }\right| v\,\textrm{d}{\boldsymbol{x}}+\epsilon ^{\frac{3}{2}}\Vert f\Vert _{H^1(\Gamma _{\epsilon };\omega _{\epsilon })}\Vert v\Vert _{H^1(\Gamma _{\epsilon };\omega _{\epsilon })}+\int _{D_{\epsilon }} gv|\nabla \omega _{\epsilon }|\,\textrm{d}{\boldsymbol{x}}. \end{aligned} \end{aligned}$$

Applying with Theorem 5, we know that

$$\begin{aligned} \begin{aligned} \int _{D_{\epsilon }} gv|\nabla \omega _{\epsilon }|\,\textrm{d}{\boldsymbol{x}}-\int _{D_{\epsilon }}{\boldsymbol{n}}\cdot (A\nabla u)v|\nabla \omega _{\epsilon }|\,\textrm{d}{\boldsymbol{x}}&=\int _{D_{\epsilon }}\left( g-{\boldsymbol{n}}\cdot (A\nabla u) \right) v|\nabla \omega _{\epsilon }|\,\textrm{d}{\boldsymbol{x}}\\&\lesssim \epsilon ^{\frac{3}{2}}\Vert {\boldsymbol{n}}\cdot (A\nabla u)-g\Vert _{H^2(\Gamma _{\epsilon };\omega _{\epsilon })}\Vert v\Vert _{H^1(D_{\epsilon };\omega _{\epsilon })}. \end{aligned} \end{aligned}$$

Therefore, we have by (26) and (27) that

27 $$\begin{aligned} \begin{aligned}&\left( u_t^{\epsilon }-u_t,v\right) _{D_{\epsilon };\omega _{\epsilon }}+a^{\epsilon }(u^{\epsilon }-u,v)\\&\quad \lesssim \epsilon ^{\frac{3}{2}}\Vert u_t\Vert _{H^1(\Gamma _{\epsilon };\omega _{\epsilon })}\Vert v\Vert _{H^1(\Gamma _{\epsilon };\omega _{\epsilon })}+\epsilon ^{\frac{3}{2}}\left\| \,\textrm{div}\left( A\nabla u\right) \right\| _{H^1(\Gamma _{\epsilon };\omega _{\epsilon })}\Vert v\Vert _{H^1(\Gamma _{\epsilon };\omega _{\epsilon })}\\&\qquad +\epsilon ^{\frac{3}{2}}\Vert {\boldsymbol{n}}\cdot (A\nabla u)-g\Vert _{H^2(\Gamma _{\epsilon };\omega _{\epsilon })}\Vert v\Vert _{H^1(D_{\epsilon };\omega _{\epsilon })}+\epsilon ^{\frac{3}{2}}\Vert f\Vert _{H^1(\Gamma _{\epsilon };\omega _{\epsilon })}\Vert v\Vert _{H^1(D_{\epsilon };\omega _{\epsilon })}. \end{aligned} \end{aligned}$$

Since $|\Gamma _{\epsilon }|\lesssim \epsilon$, applying $v=u^{\epsilon }-u$ to (27) gives

$$\begin{aligned} \begin{aligned}&\frac{1}{2}\frac{\,\textrm{d}}{\,\textrm{d}t}\Vert u^{\epsilon }-u\Vert _{L^2(D_{\epsilon };\omega _{\epsilon })}^2+\Vert \nabla (u^{\epsilon }-u)\Vert _{L^2(D_{\epsilon };\omega _{\epsilon })}^2\\&\quad \lesssim \epsilon ^{\frac{3}{2}}\Big (\Vert u_t\Vert _{H^1(\Gamma _{\epsilon };\omega _{\epsilon })}+\Vert \,\textrm{div}(A\nabla u)\Vert _{H^1(\Gamma _{\epsilon };\omega _{\epsilon })}+\Vert {\boldsymbol{n}}\cdot (A\nabla u)-g\Vert _{H^2(\Gamma _{\epsilon };\omega _{\epsilon })}\\&\qquad +\Vert f\Vert _{H^1(\Gamma _{\epsilon };\omega _{\epsilon })}\Big )\Vert u^{\epsilon }-u\Vert _{H^1(\Gamma _{\epsilon };\omega _{\epsilon })}\\&\quad \lesssim \epsilon ^4 +\Vert u^{\epsilon }-u\Vert _{L^2(D_{\epsilon };\omega _{\epsilon })}^2+\Vert \nabla (u^{\epsilon }-u)\Vert _{L^2(D_{\epsilon };\omega _{\epsilon })}^2. \end{aligned} \end{aligned}$$

Thus

$$\begin{aligned} \frac{1}{2}\frac{\,\textrm{d}}{\,\textrm{d}t}\Vert u^{\epsilon }-u\Vert _{L^2(D_{\epsilon };\omega _{\epsilon })}^2 \lesssim \epsilon ^4+\Vert u^{\epsilon }-u\Vert _{L^2(D_{\epsilon };\omega _{\epsilon })}^2, \end{aligned}$$

which implies

$$\begin{aligned} \Vert u^{\epsilon }-u\Vert _{L^2(D_{\epsilon };\omega _{\epsilon })}\lesssim \epsilon ^2. \end{aligned}$$

The proof is completed.

$$\square $$

### Optimal error estimate in the $H^1$-norm

First we derive several important lemmas needed for the $H^1$-norm error estimate.

#### Lemma 2

Suppose that $0<\epsilon \le \epsilon _0$, $g(t)\in H^1(D_{\epsilon };\omega _{\epsilon })$, and *f* satisfies Assumption 1. Assume the exact solution $u\in L^2(0,T;H^1(D))$ and its extension to $D_\epsilon$ fulfills Assumption 2. Then we have

28 $$\begin{aligned} \int _{\partial D}g(u^{\epsilon }-u)\,\textrm{d}\sigma +\int _{D_{\epsilon }} g|\nabla \omega _{\epsilon }|(u^{\epsilon }-u)\,\textrm{d}{\boldsymbol{x}}\le C\epsilon ^2+\frac{1}{4}\Vert \nabla u^{\epsilon }-\nabla u\Vert _{L^2(\Gamma _{\epsilon };\omega _{\epsilon })}^2, \end{aligned}$$

where *C* is a constant independent of $\epsilon$ but linearly dependent of *D*.

#### Proof

Using $\nabla d_D({\boldsymbol{x}})={\boldsymbol{n}}({\boldsymbol{x}})$, $\nabla \omega _{\epsilon }=-{\boldsymbol{n}}|\nabla \omega _{\epsilon }|$ and the divergence theorem, we derive that

29 $$\begin{aligned} \begin{aligned}&\int _{\partial D}g(u^{\epsilon }-u)\,\textrm{d}\sigma + \int _{D_{\epsilon }}g|\nabla \omega _{\epsilon }|(u^{\epsilon }-u)\,\textrm{d}{\boldsymbol{x}}\\&\quad =\int _{\partial D}g(u^{\epsilon }-u)\,\textrm{d}\sigma -\int _{\partial D_{\epsilon }}g|\nabla \omega _{\epsilon }|(u^{\epsilon }-u)\,\textrm{d}{\boldsymbol{x}}\\&\quad =\int _D \,\textrm{div}(g\nabla d_D(u^{\epsilon }-u))\,\textrm{d}{\boldsymbol{x}}-\int _{D_{\epsilon }}\,\textrm{div}(g\nabla d_D(u^{\epsilon }-u))\omega _{\epsilon }\,\textrm{d}{\boldsymbol{x}}\\&\quad =\left| \int _D \,\textrm{div}(g\nabla d_D)\left( u^{\epsilon }-u\right) \,\textrm{d}{\boldsymbol{x}}-\int _{D_{\epsilon }}\,\textrm{div}(g\nabla d_D)\left( u^{\epsilon }-u\right) \omega _{\epsilon } \,\textrm{d}{\boldsymbol{x}}\right| \\&\qquad +\left| \int _D g\nabla d_D\cdot \left( \nabla u^{\epsilon }-\nabla u\right) \,\textrm{d}{\boldsymbol{x}}-\int _{D_{\epsilon }}g\nabla d_D\cdot \left( \nabla u^{\epsilon }-\nabla u\right) \omega _{\epsilon }\,\textrm{d}{\boldsymbol{x}}\right| \\&\quad =:\textrm{II}_1+\textrm{II}_2. \end{aligned} \end{aligned}$$

By using the conclusion of Lemma 1 and $|\Gamma _{\epsilon }|\lesssim \epsilon$, we obtain

30 $$\begin{aligned} \left\{ \begin{aligned} \textrm{II}_1&\lesssim \left\| \,\textrm{div}(g\nabla d_D)\right\| _{L^2(\Gamma _{\epsilon };\omega _{\epsilon })}\epsilon ^{\frac{1}{2}}\Vert u^{\epsilon }-u\Vert _{L^2(\Gamma _{\epsilon };\omega _{\epsilon })}\le C\epsilon ^2+\frac{1}{8}\Vert u^{\epsilon }-u\Vert _{L^2(\Gamma _{\epsilon };\omega _{\epsilon })}^2,\\ \textrm{II}_2&\lesssim \Vert g\Vert _{L^2(\Gamma _{\epsilon };\omega _{\epsilon })}\epsilon ^{\frac{1}{2}}\Vert \nabla u^{\epsilon }-\nabla u\Vert _{L^2(\Gamma _{\epsilon };\omega _{\epsilon })} \le C\epsilon ^2+\frac{1}{8}\Vert \nabla u^{\epsilon }-\nabla u\Vert _{L^2(\Gamma _{\epsilon };\omega _{\epsilon })}^2. \end{aligned}\right. \end{aligned}$$

Combining (30) and (29) together, and applying the $L^2$ norm error estimate in Theorem 6, we easily derive the result (28).

$$\square $$

#### Lemma 3

Suppose that $0<\epsilon \le \epsilon _0$, $g\in L^{\infty }(0,T;H^1(D_{\epsilon };\omega _{\epsilon }))$, and $g_t\in L^{\infty }(0,T;L^{\infty }(D_{\epsilon }))$. Assume the exact solution $u\in L^2(0,T;H^1(D))$ and its extension to $D_\epsilon$ fulfills Assumption 2. If $\kappa \le A({\boldsymbol{x}}) \le \kappa ^{-1}$ for all ${\boldsymbol{x}} \in D_{\epsilon }$ with some constant $\kappa >0$, then we have

31 $$\begin{aligned}  &   \Big |\int _0^T\int _{D_{\epsilon }}g(u_t^{\epsilon }-u_t)|\nabla \omega _{\epsilon }|\,\textrm{d}{\boldsymbol{x}}\,\textrm{d}t-\int _0^T \int _{D_{\epsilon }} A\nabla u_t\cdot \nabla \omega _{\epsilon }(u^{\epsilon }-u)\,\textrm{d}{\boldsymbol{x}}\,\textrm{d}t\nonumber \\  &   -\int _0^T\int _{\partial D}g(u_t^{\epsilon }-u_t)\,\textrm{d}\sigma \,\textrm{d}t -\int _0^T\int _{\partial D}g_t(u^{\epsilon }-u)\,\textrm{d}\sigma \,\textrm{d}t\Big |\nonumber \\  &   \qquad \le C\epsilon ^2+\frac{1}{4}\Vert \nabla u^{\epsilon }(T,\cdot )-\nabla u(T,\cdot )\Vert _{L^2(\Gamma _{\epsilon };\omega _{\epsilon })}^2+\frac{1}{4}\Vert \nabla u^{\epsilon }(0,\cdot )-\nabla u(0,\cdot )\Vert _{L^2(\Gamma _{\epsilon };\omega _{\epsilon })}^2,\nonumber \\ \end{aligned}$$

where *C* is a constant independent of $\epsilon$ but linearly dependent of *D* and *T*.

#### Proof

Applying with integration by part, we can reformulate the left-hand side of (31) as

32 $$\begin{aligned} \begin{aligned}&\Big |\int _0^T\int _{D_{\epsilon }}g(u_t^{\epsilon }-u_t)|\nabla \omega _{\epsilon }|\,\textrm{d}{\boldsymbol{x}}\,\textrm{d}t-\int _0^T \int _{D_{\epsilon }} A\nabla u_t\cdot \nabla \omega _{\epsilon }(u^{\epsilon }-u)\,\textrm{d}{\boldsymbol{x}}\,\textrm{d}t\\&-\int _0^T\int _{\partial D}g(u_t^{\epsilon }-u_t)\,\textrm{d}\sigma \,\textrm{d}t-\int _0^T\int _{\partial D}g_t(u^{\epsilon }-u)\,\textrm{d}\sigma \,\textrm{d}t\Big |\\&\quad \le \left| \int _{D_{\epsilon }}g(T,\cdot )(u^{\epsilon }(T,\cdot )-u(T,\cdot ))|\nabla \omega _{\epsilon }|\,\textrm{d}{\boldsymbol{x}}-\int _{\partial D}g(T,\cdot )(u^{\epsilon }(T,\cdot )-u(T,\cdot ))\,\textrm{d}\sigma \right| \\&\qquad +\left| \int _{D_{\epsilon }}g(0,\cdot )(u^{\epsilon }(0,\cdot )-u(0,\cdot ))|\nabla \omega _{\epsilon }|\,\textrm{d}{\boldsymbol{x}}-\int _{\partial D}g(0,\cdot )(u^{\epsilon }(0,\cdot )-u(0,\cdot ))\,\textrm{d}\sigma \right| \\&\qquad +\left| \int _0^T\int _{D_{\epsilon }}g_t(u^{\epsilon }-u)|\nabla \omega _{\epsilon }|\,\textrm{d}{\boldsymbol{x}}\,\textrm{d}t+\int _0^T\int _{D_{\epsilon }}A\nabla u_t\cdot \nabla \omega _{\epsilon }(u^{\epsilon }-u)\,\textrm{d}{\boldsymbol{x}}\,\textrm{d}t \right| \\&\quad =:\textrm{III}_1+\textrm{III}_2+\textrm{III}_3. \end{aligned} \end{aligned}$$

In the remaining part, we will estimate the term $\textrm{III}_1$, $\textrm{III}_2$ and $\textrm{III}_3$, respectively. As for the estimate of $\textrm{III}_1$ and $\textrm{III}_2$, we can apply the Lemma 2 to derive

33 $$\begin{aligned} \left\{ \begin{aligned} \textrm{III}_1&\le C\epsilon ^2+\frac{1}{4}\Vert \nabla u^{\epsilon }(T,\cdot )-\nabla u(T,\cdot )\Vert _{L^2(\Gamma _{\epsilon };\omega _{\epsilon })}^2,\\ \mathrm {III_2}&\le C\epsilon ^2+\frac{1}{4} \Vert \nabla u^{\epsilon }(0,\cdot )-\nabla u(0,\cdot )\Vert _{L^2(\Gamma _{\epsilon };\omega _{\epsilon })}^2. \end{aligned}\right. \end{aligned}$$

As for the estimate of $\textrm{III}_3$, by using the Cauchy-Schwarz inequality and the definition of $\omega _{\epsilon }$, we have

34 $$\begin{aligned} \begin{aligned} \textrm{III}_3&= \left| \int _0^T\int _{D_{\epsilon }} g_t(u^{\epsilon }-u)|\nabla \omega _{\epsilon }|\,\textrm{d}{\boldsymbol{x}}\,\textrm{d}t-\int _0^T\int _{D_{\epsilon }}{\boldsymbol{n}} \cdot (A\nabla u_t)(u^{\epsilon }-u)|\nabla \omega _{\epsilon }|\,\textrm{d}{\boldsymbol{x}}\,\textrm{d}t \right| \\&=\left| \int _0^T\int _{D_{\epsilon }}({\boldsymbol{n}}\cdot (A\nabla u_t)-g_t)(u^{\epsilon }-u)|\nabla \omega _{\epsilon }|\,\textrm{d}{\boldsymbol{x}}\,\textrm{d}t \right| \\&\le \int _0^T\left( \int _{D_{\epsilon }}\left| \omega _{\epsilon }^{\frac{1}{2}}(u^{\epsilon }-u) \right| ^2\,\textrm{d}{\boldsymbol{x}} \right) ^{\frac{1}{2}}\left( \int _{D_{\epsilon }}\left| ({\boldsymbol{n}}\cdot (A\nabla u_t)-g_t)\left| \nabla \omega _{\epsilon }\right| /\omega _{\epsilon }^{\frac{1}{2}} \right| ^2\,\textrm{d}{\boldsymbol{x}}\right) ^{\frac{1}{2}}\,\textrm{d}t\\&\lesssim \sup _{0\le t\le T}\Vert u^{\epsilon }(t)-u(t)\Vert _{L^2(D_{\epsilon };\omega _{\epsilon })}\Vert {\boldsymbol{n}}\cdot (A\nabla u_t)-g_t\Vert _{L^{\infty }(D_{\epsilon })}\\&\lesssim \epsilon ^2. \end{aligned} \end{aligned}$$

Inserting (33) and (34) to (32), we finally obtain (31), which completes the proof.

$$\square $$

#### Theorem 7

(Error estimate in the $H^1$-norm) Suppose that $0<\epsilon \le \epsilon _0$, $g\in L^{\infty }(0,T;H^2(D_{\epsilon };\omega _{\epsilon }))$, $g_t\in L^{\infty }(0,T;L^{\infty }(D_{\epsilon }))$, $\kappa \le A({\boldsymbol{x}})\le \kappa ^{-1}$ for all ${\boldsymbol{x}} \in D$ with some constant $\kappa >0$, and *f* satisfies Assumption 1. Assume the exact solution $u\in L^2(0,T;H^1(D_{\epsilon }))$ and its extension to $D_\epsilon$ fulfills Assumption 2. Then we have

35 $$\begin{aligned} \Vert u^{\epsilon }(t)-u(t)\Vert _{H^1(D_{\epsilon };\omega _{\epsilon })}\lesssim \epsilon , \quad \forall \ 0 \le t\le T, \end{aligned}$$

where the hidden constant is independent of $\epsilon$, and linearly dependent of *D* and *T*.

#### Proof

First, we insert $v=2(u^{\epsilon }_t-u_t)$ into (19), we have

36 $$\begin{aligned} \begin{aligned}&2\left( u_t^{\epsilon }-u_t,u_t^{\epsilon }-u_t\right) _{D_{\epsilon },\omega _{\epsilon }}+2a^{\epsilon }(u^{\epsilon }-u,u_t^{\epsilon }-u_t)\\&\quad = 2\left( u_t,u_t^{\epsilon }-u_t\right) -2\left( u_t,u_t^{\epsilon }-u_t\right) +2a(u,u_t^{\epsilon }-u_t)-2a^{\epsilon }(u,u_t^{\epsilon }-u_t)\\&\quad \quad +2\ell ^{\epsilon }(u_t^{\epsilon }-u_t)-2\ell (u_t^{\epsilon }-u_t). \end{aligned} \end{aligned}$$

Then, after integrating both sides of (36) with respect to *t*, we can derive that

37 $$\begin{aligned} \begin{aligned}&\int _0^T2\left\| u_t^{\epsilon }-u_t\right\| _{L^2(D_{\epsilon };\omega _{\epsilon })}^2+\frac{\,\textrm{d}}{\,\textrm{d}t}a^{\epsilon }(u^{\epsilon }-u,u^{\epsilon }-u)\,\textrm{d}t\\&\quad \le \int _0^T\left( u_t,u_t^{\epsilon }-u_t\right) -\left( u_t,u_t^{\epsilon }-u_t\right) _{D_{\epsilon };\omega _{\epsilon }}\,\textrm{d}t+2\int _0^T a(u,u_t^{\epsilon }-u_t)-a^{\epsilon }(u,u_t^{\epsilon }-u_t)\,\textrm{d}t\\&\quad \quad +2\int _0^T\ell ^{\epsilon }(u_t^{\epsilon }-u_t)-\ell (u_t^{\epsilon }-u_t)\,\textrm{d}t\\&\quad =:\textrm{IV}_1+\textrm{IV}_2+\textrm{IV}_3. \end{aligned} \end{aligned}$$

Next, we will estimate $\textrm{IV}_1$, $\textrm{IV}_2$ and $\textrm{IV}_3$ respectively. For $\textrm{IV}_1$, applying with Lemma 1, we have

$$\begin{aligned} \begin{aligned} \left| \textrm{IV}_1\right|&= 2\left| \int _0^T \left( u_t,u_t^{\epsilon }-u_t\right) -\left( u_t,u_t^{\epsilon }-u_t\right) _{D_{\epsilon };\omega _{\epsilon }} \,\textrm{d}t\right| \\&\lesssim \int _0^T\left| \left( u_t,u_t^{\epsilon }-u_t\right) -\left( u_t,u_t^{\epsilon }-u_t\right) _{D_{\epsilon };\omega _{\epsilon }} \right| \,\textrm{d}t\\&\lesssim \int _0^T\left\| u_t^{\epsilon }-u_t\right\| _{L^2(\Gamma _{\epsilon };\omega _{\epsilon })}\epsilon ^{\frac{1}{2}}\Vert u_t\Vert _{L^2(\Gamma _{\epsilon };\omega _{\epsilon })}\,\textrm{d}t. \end{aligned} \end{aligned}$$

Due to the fact that $u^{\epsilon }$ and $u_t^{\epsilon }$, as well as *u* and $u_t$, have the same regularity, based on Theorem 6, we get that

38 $$\begin{aligned} |\textrm{IV}_1|&\lesssim \int _0^T \epsilon ^2\epsilon ^{\frac{1}{2}}\Vert u_t\Vert _{\Gamma _{\epsilon };\omega _{\epsilon }}\,\textrm{d}t\lesssim \epsilon ^3. \end{aligned}$$

As for $\textrm{IV}_2$, with the integration by part, we derive that

39 $$\begin{aligned} \begin{aligned} \textrm{IV}_2=&\,2\int _0^T a(u,u_t^{\epsilon }-u_t)-a^{\epsilon }(u,u_t^{\epsilon }-u_t)\,\textrm{d}t \\ =&\,2\int _D A\Big ( \nabla u(T)\cdot \left( \nabla u^{\epsilon }(T)-\nabla u(T)\right) -\nabla u(0)\cdot \left( \nabla u^{\epsilon } (0)-\nabla u(0)\right) \\&-\int _0^T (\nabla u^{\epsilon }-\nabla u)\cdot \nabla u_t\,\textrm{d}t\Big )\,\textrm{d}{\boldsymbol{x}}-2\int _D A\Big ( \nabla u(T)\cdot \left( \nabla u^{\epsilon }(T)-\nabla u(T)\right) \\&-\nabla u(0)\cdot \left( \nabla u^{\epsilon }(0)-\nabla u(0)\right) -\int _0^T (\nabla u^{\epsilon }-\nabla u)\cdot \nabla u_t\,\textrm{d}t\Big )\,\textrm{d}{\boldsymbol{x}}\\ =&\,2\Big (\int _DA\nabla u(T)\cdot (\nabla u^{\epsilon }(T)-\nabla u(T))\,\textrm{d}{\boldsymbol{x}}-\int _{D_{\epsilon }} A\nabla u(T)\\&\cdot (\nabla u^{\epsilon }(T)-\nabla u(T))\omega _{\epsilon }\,\textrm{d}{\boldsymbol{x}}\Big ) +2\Big (\int _{D_{\epsilon }}A\nabla u(0)\cdot (\nabla u^{\epsilon }(0)-\nabla u(0))\omega _{\epsilon }\,\textrm{d}{\boldsymbol{x}}\\&-\int _D A\nabla u(0)\cdot (\nabla u^{\epsilon }(0)-\nabla u(0))\,\textrm{d}{\boldsymbol{x}}\Big ) +2\Big (\int _0^T\Big (\int _{D_{\epsilon }} A(\nabla u^{\epsilon }-\nabla u)\\&\cdot \nabla u_t\omega _{\epsilon }\,\textrm{d}{\boldsymbol{x}}-\int _D A(\nabla u^{\epsilon }-\nabla u)\cdot \nabla u_t\,\textrm{d}{\boldsymbol{x}}\Big )\,\textrm{d}t\Big )\\ =:&\,\textrm{V}_1+\textrm{V}_2+\textrm{V}_3. \end{aligned} \end{aligned}$$

For $\textrm{V}_1$, applying with Lemma 1, we know that

40 $$\begin{aligned} \begin{aligned} |\textrm{V}_1|&\lesssim \epsilon ^{\frac{1}{2}}\Vert \nabla u^{\epsilon }(T)-\nabla u(T)\Vert _{L^2(\Gamma _{\epsilon };\omega _{\epsilon })}\Vert A\nabla u(T)\Vert _{L^2(\Gamma _{\epsilon };\omega _{\epsilon })}\\&\le \frac{1}{4} \Vert \nabla u^{\epsilon }(T)-\nabla u(T)\Vert _{L^2(\Gamma _{\epsilon };\omega _{\epsilon })}^2+C\epsilon \Vert A\nabla u(T)\Vert _{L^2(\Gamma _{\epsilon };\omega _{\epsilon })}^2\\&\le \frac{1}{4} \Vert \nabla u^{\epsilon }(T)-\nabla u(T)\Vert _{L^2(\Gamma _{\epsilon };\omega _{\epsilon })}^2+C\epsilon ^2, \end{aligned} \end{aligned}$$

Similar as the derivation of (40), we have

41 $$\begin{aligned} |\textrm{V}_2|&\lesssim \epsilon ^\frac{1}{2}\Vert \nabla u^{\epsilon }(0)-\nabla u(0)\Vert _{L^2(\Gamma _{\epsilon };\omega _{\epsilon })}\Vert A\nabla u(0)\Vert _{L^2(\Gamma _{\epsilon };\omega _{\epsilon })}\lesssim \epsilon ^2, \end{aligned}$$

with proper initial guess for $u^{\epsilon }$. For the remaining term $\textrm{V}_3$, applying with the variational formula and the property of $\omega _{\epsilon }$, we can rewrite $\textrm{V}_3$ as

42 $$\begin{aligned} \textrm{V}_3= &   \,2\left( \int _0^T\left( \int _{D_{\epsilon }} A(\nabla u^{\epsilon }-\nabla u)\cdot \nabla u_t\omega _{\epsilon }\,\textrm{d}{\boldsymbol{x}}-\int _D A(\nabla u^{\epsilon }-\nabla u)\cdot \nabla u_t\,\textrm{d}{\boldsymbol{x}}\right) \,\textrm{d}t\right) \nonumber \\= &   \,2\int _0^T\int _{\partial D_{\epsilon }}{\boldsymbol{n}}\cdot (A\nabla u_t)\omega _{\epsilon }(u^{\epsilon }-u)\,\textrm{d}\sigma -\int _{D_{\epsilon }}\,\textrm{div}(A\nabla u_t\omega _{\epsilon })(u^{\epsilon }-u)\,\textrm{d}{\boldsymbol{x}}\nonumber \\  &   -\int _{\partial D}{\boldsymbol{n}}\cdot (A\nabla u_t)(u^{\epsilon }-u)\,\textrm{d}\sigma +\int _D \,\textrm{div}(A\nabla u_t)(u^{\epsilon }-u)\,\textrm{d}{\boldsymbol{x}}\,\textrm{d}t\nonumber \\= &   \,2\left( \int _0^T\int _D \,\textrm{div}(A\nabla u_t)(u^{\epsilon }-u)\,\textrm{d}{\boldsymbol{x}}-\int _{D_{\epsilon }}\,\textrm{div}(A\nabla u_t)(u^{\epsilon }-u)\omega _{\epsilon }\,\textrm{d}{\boldsymbol{x}}\,\textrm{d}t\right) \nonumber \\  &   -2\left( \int _0^T\int _{D_{\epsilon }} A\nabla u_t\cdot \nabla \omega _{\epsilon }(u^{\epsilon }-u)\,\textrm{d}{\boldsymbol{x}}+\int _{\partial D}g_t(u^{\epsilon }-u)\,\textrm{d}\sigma \,\textrm{d}t\right) \nonumber \\=: &   \,\textrm{W}_1-\textrm{W}_2. \end{aligned}$$

Following the similar derivation of (40), we obtain

43 $$\begin{aligned} \begin{aligned} |\textrm{W}_1|&\lesssim \epsilon ^{\frac{1}{2}}\Vert u^{\epsilon }-u\Vert _{L^2(\Gamma _{\epsilon };\omega _{\epsilon })}\Vert \,\textrm{div}(A\nabla u_t)\Vert _{L^2(\Gamma _{\epsilon };\omega _{\epsilon })}\lesssim \epsilon ^2. \end{aligned} \end{aligned}$$

The terms remained to be analyzed are $\textrm{W}_2$ and $\textrm{IV}_3$. Merging $\textrm{W}_2$ and $\textrm{IV}_3$ together, we have

44 $$\begin{aligned} \begin{aligned} |-\textrm{W}_2+\textrm{IV}_3|\le&\,2\left| \int _0^T\int _{D_{\epsilon }}f(u_t^{\epsilon }-u_t)\omega _{\epsilon }\,\textrm{d}{\boldsymbol{x}}-\int _D f(u_t^{\epsilon }-u_t)\,\textrm{d}{\boldsymbol{x}}\,\textrm{d}t\right| \\&+2\left| \int _0^T\int _{D_{\epsilon }} g(u_t^{\epsilon }-u_t)|\nabla \omega _{\epsilon }|\,\textrm{d}{\boldsymbol{x}}-\int _{\partial D} g(u_t^{\epsilon }-u_t)\,\textrm{d}\sigma \,\textrm{d}t\right. \\&\left. -\int _0^T\int _{D_{\epsilon }} A\nabla u_t\cdot \nabla \omega _{\epsilon }(u^{\epsilon }-u)\,\textrm{d}{\boldsymbol{x}}+\int _{\partial D}g_t(u^{\epsilon }-u)\,\textrm{d}\sigma \,\textrm{d}t\right| . \end{aligned} \end{aligned}$$

By using the Lemma 1, we can easily derive that

45 $$\begin{aligned} \begin{aligned}&\left| \int _0^T\int _{D_{\epsilon }}f(u_t^{\epsilon }-u_t)\omega _{\epsilon }\,\textrm{d}{\boldsymbol{x}}-\int _D f(u_t^{\epsilon }-u_t)\,\textrm{d}{\boldsymbol{x}}\,\textrm{d}t\right| \\&\qquad \lesssim \int _0^T \epsilon ^{\frac{1}{2}}\Vert f\Vert _{L^2(\Gamma _{\epsilon };\omega _{\epsilon })}\Vert u_t^{\epsilon }-u_t\Vert _{L^2(\Gamma _{\epsilon };\omega _{\epsilon })}\,\textrm{d}t\lesssim \epsilon ^3. \end{aligned} \end{aligned}$$

By using the Lemma 3, we can derive that

46 $$\begin{aligned} \begin{aligned}&\left| \int _0^T\int _{D_{\epsilon }} g(u_t^{\epsilon }-u_t)|\nabla \omega _{\epsilon }|\,\textrm{d}{\boldsymbol{x}}\,\textrm{d}t-\int _0^T\int _{D_{\epsilon }}A\nabla u_t\cdot \nabla \omega _{\epsilon }(u^{\epsilon }-u)\,\textrm{d}{\boldsymbol{x}}\,\textrm{d}t\right. \\&\left. -\int _0^T\int _{\partial D}g(u_t^{\epsilon }-u_t)\,\textrm{d}\sigma \,\textrm{d}t -\int _0^T\int _{\partial D} g_t(u^{\epsilon }-u)\,\textrm{d}\sigma \,\textrm{d}t\right| \\&\qquad \le C\epsilon ^2+\frac{1}{4}\Vert \nabla u^{\epsilon }(T)-\nabla u(T)\Vert _{L^2(\Gamma _{\epsilon };\omega _{\epsilon })}^2+\frac{1}{4} \Vert \nabla u^{\epsilon }(0)-\nabla u(0)\Vert _{L^2(\Gamma _{\epsilon };\omega _{\epsilon })}^2. \end{aligned} \end{aligned}$$

Combining (45) and (46) together, we can reformulate (44) as

47 $$\begin{aligned} \begin{aligned} |-\textrm{W}_2+\textrm{IV}_3|\le&\, C\epsilon ^2+\frac{1}{4}\Vert \nabla u^{\epsilon }(T)-\nabla u(T)\Vert _{L^2(\Gamma _{\epsilon };\omega _{\epsilon })}^2\\&\,+\frac{1}{4} \Vert \nabla u^{\epsilon }(0)-\nabla u(0)\Vert _{L^2(\Gamma _{\epsilon };\omega _{\epsilon })}^2. \end{aligned} \end{aligned}$$

Therefore, inserting (38)-(47) to (37), we can conclude that

$$\begin{aligned} \begin{aligned}&\int _0^T 2\Vert u_t^{\epsilon }-u_t\Vert _{L^2(D_{\epsilon };\omega _{\epsilon })}^2\,\textrm{d}t+\Vert \nabla u^{\epsilon }(T)-\nabla u(T)\Vert _{L^2(D_{\epsilon };\omega _{\epsilon })}^2\\&\qquad -\Vert \nabla u^{\epsilon }(0)-\nabla u(0)\Vert _{L^2(D_{\epsilon };\omega _{\epsilon })}^2\\&\qquad \le C\epsilon ^2+\frac{1}{2}\Vert \nabla u^{\epsilon }(T)-\nabla u(T)\Vert _{L^2(D_{\epsilon };\omega _{\epsilon })}^2+\frac{1}{4}\Vert \nabla u^{\epsilon }(0)-\nabla u(0)\Vert _{L^2(D_{\epsilon };\omega _{\epsilon })}^2, \end{aligned} \end{aligned}$$

which implies that

$$\begin{aligned} \Vert \nabla u^{\epsilon }(T)-\nabla u(T)\Vert _{L^2(D_{\epsilon };\omega _{\epsilon })}^2\lesssim \epsilon ^2 + \Vert \nabla u^{\epsilon }(0)-\nabla u(0)\Vert _{L^2(D_{\epsilon };\omega _{\epsilon })}^2. \end{aligned}$$

Since *T* can be replaced by any time $t\in [0,T]$ in the above derivations, we obtain (35).

$$\square $$

#### Remark 1

In this work, we focus only on the case of Neumann boundary conditions. For Robin boundary conditions, the design of the DDM scheme is very similar to the Neumann case, and the corresponding error estimates can be obtained with minor modifications from [15]. In contrast, the Dirichlet case is quite different, as the boundary values are fixed and typically require additional constraints or penalty terms. In [15], the original problem is reformulated as a constrained optimization problem, and the analysis is based on optimization theory and error estimates for both volume and boundary integrals. In [39], penalty terms are introduced, and an asymptotic analysis of the boundary layers is developed.

#### Remark 2

We only need analyze the error estimates on the extended domain $D_{\epsilon }$. As for the error estimates on the physical domain *D*, since $\omega _{\epsilon } \ge 1/2$ for any ${\boldsymbol{x}} \in D$, we can easily derived that, under the same assumptions, the error estimates in the domain *D* becomes

$$\begin{aligned} \left\| u^{\epsilon }(t)-u(t)\right\| _{L^2(D)}\lesssim \epsilon ^2, \quad \left\| u^{\epsilon }(t)-u(t)\right\| _{H^1(D)}\lesssim \epsilon , \quad \forall \ 0 \le t \le T. \end{aligned}$$

#### Remark 3

This paper mainly focuses on the approximate errors between the original PDE problem defined on irregular domain and the transformed PDE problem produced by the diffuse domain method. The interface thickness $\epsilon$ and the further numerical discretization method can be chosen arbitrarily based on the physical domain and the practical needs. It is worthy to note that the spatial mesh size should be significantly smaller (at least 4 times) than $\epsilon$, so that the numerical errors will be dominated by the DDM approximation errors and the effect of interface thickness on the DDM accuracy can be easily captured. On the other hand, as $\epsilon$ becomes smaller, the transition zone becomes narrower and meshes needed for numerical discretization also become finer, and the resulting fully-discrete system of the problem will become more difficult to solve in practice.

## Numerical experiments

In this section, we will present some numerical experiments to verify the error estimates (Theorems 6 and 7 ) obtained in Section 4 and demonstrate the performance of the DDM. We apply the finite element method with the bilinear basis functions for space discretization and the BDF2 scheme for time stepping to solve the DDM-transformed problem (10) defined on a larger rectangular domain, which is of second-order in time, and first-order in space with respect to the $H^1$ norm and second-order in space with respect to the $L^2$ norm. To demonstrate the convergence order of the diffuse domain solution with respect to the interface width parameter $\epsilon$, i.e., $\left\| u(t)-u^{\epsilon }(t)\right\| _{L^2(D_{\epsilon };\omega _{\epsilon })}$ and $\left\| u(t)-u^{\epsilon }(t)\right\| _{H^1(D_{\epsilon };\omega _{\epsilon })}$, we will take $\epsilon$ to be much larger than the spatial mesh size and the time step size (so that the solution errors are mainly caused by the DDM approximation). For simplicity, we only evaluate the approximation error on the original domain *D*, i.e., $\left\| u(t)-u^{\epsilon }(t)\right\| _{L^2(D;\omega _{\epsilon })}$ and $\left\| u(t)-u^{\epsilon }(t)\right\| _{H^1(D;\omega _{\epsilon })}$. All tests are done using Matlab on a laptop with Intel Ultra 9 185H, 2.30GHz CPU and 32GB memory.

**Table 1:** Numerical results on the solution errors measured in the weighted $L^2$ and $H^1$ norms and corresponding convergence rates at the terminal time $T=0.5$ produced by the DDM in Example 1

$\epsilon$	$N_x\times N_y$	$N_T$	$\Vert u^{\epsilon }-u(t_n)\Vert _{L^2(D;\omega _{\epsilon })}$	CR	$\Vert u^{\epsilon }-u(t_n)\Vert _{H^1(D;\omega _{\epsilon })}$	CR
Convergence tests for the circular domain						
1/8	$512\times 512$	512	1.0000e-03	-	1.0000e-03	-
1/16	$512\times 512$	512	2.6803e-04	1.90	2.7212e-04	1.88
1/32	$512\times 512$	512	6.8145e-05	1.98	7.6650e-05	1.83
1/64	$512\times 512$	512	1.7663e-05	1.95	3.8208e-05	1.00
Convergence tests for the flower-shaped domain						
1/8	$512\times 512$	512	7.7428e-04	-	7.8951e-04	-
1/16	$512\times 512$	512	2.1302e-04	1.86	2.1599e-04	1.87
1/32	$512\times 512$	512	5.4320e-05	1.97	5.9805e-05	1.85
1/64	$512\times 512$	512	1.3876e-05	1.97	2.7572e-05	1.12

**Fig. 2:**
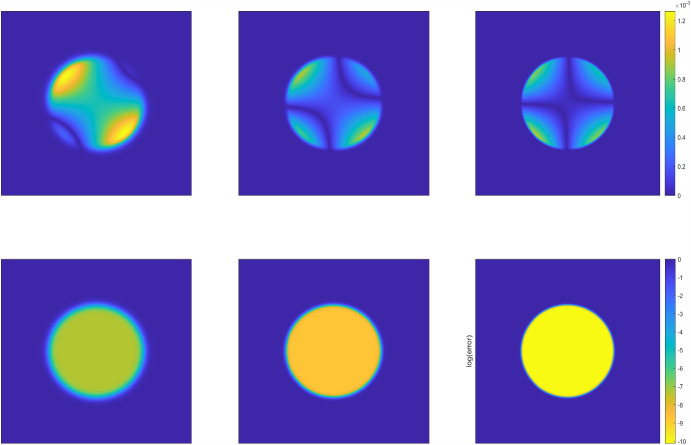
The phase structures of the numerical solutions (top row) and the corresponding numerical error (bottom row) at the terminal time $T=0.5$ produced by the DDM approach with interface thickness $\epsilon =1/16,1/32,1/64$ (from left to right) for Example 1 in the circular domain

### The case of constant diffusion coefficient

#### Example 1

In this example, we consider the following two-dimensional diffusion problem with a constant diffusion coefficient: for $0 \le t \le T$,

48 $$\begin{aligned} \left\{ \begin{array}{ll} \,u_t=3\Delta u +f(t, x, y), \   & (x,y)\in D\\ \,u(0,x,y) = \left( x^2+2x\right) \left( y^2-2y \right) , \ \ & (x, y) \in D, \end{array} \right. \end{aligned}$$

where

$$\begin{aligned} \begin{aligned} f(t, x, y) =&-e^{-\pi ^2 t}\left( \pi ^2\left( x^2+2x\right) \left( y^2-2y\right) +6\left( x^2+2x\right) +6\left( y^2-2y\right) \right) . \end{aligned} \end{aligned}$$

The exact solution is given by

$$\begin{aligned} u(t,x,y)=e^{-\pi ^2 t}\left( x^2+2x \right) \left( y^2-2y \right) . \end{aligned}$$

Two different domains *D* are considered: one is a circular domain defined by

49 $$\begin{aligned} D=\left\{ (x,y)\;\big |\; x^2+y^2=\frac{1}{16} \right\} , \end{aligned}$$

and the other is a flower-shaped domain defined by

50 $$\begin{aligned} D=\left\{ (x,y)\;\big |\; x^2+y^2-\left( 0.175-0.03\sin \left( 4\arctan \left( \frac{y}{x}\right) \right) \right) ^2=0 \right\} . \end{aligned}$$

The Neumann boundary condition is imposed correspondingly, and the terminal time is chosen as $T=0.5$.

**Fig. 3:**
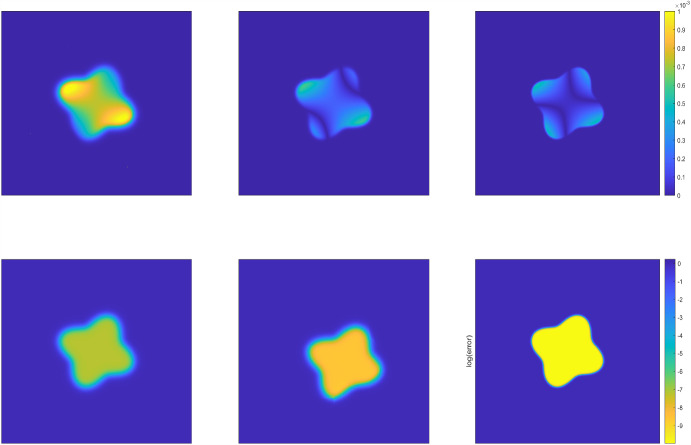
The phase structures of the numerical solutions (top row) and the corresponding numerical error (bottom row) at the terminal time $T=0.5$ produced by the DDM approach with interface thickness $\epsilon =1/16,1/32,1/64$ (from left to right) for Example 1 in the flower domain

We run the diffuse domain method (DDM) on the extended rectangular domain $\Omega =[-1/2,1/2]\times [-1/2, 1/2]$ and $D\subset \Omega$. We take the time steps $N_T=512$ (i.e., $\Delta \tau = T/N_T=1/1024$ and the uniformly spatial mesh with $h_x = h_y=1/512$. Then we set the interface thickness $\epsilon$ = 1/8, 1/16, 1/32, 1/64, respectively, so that the spatial mesh size and temporal step size are much finer than the interface thickness $\epsilon$. All numerical results are reported in Table 1, including the solution errors measured in the weighted $L^2$ and $H^1$ norms and the corresponding convergence rates. We observe roughly second-order convergence rates with respect to the weighted $L^2$ norm and first-order convergence rates with respect to the weighted $H^1$ norm as expected, which coincide very well with the error estimates derived in Theorems 6 and 7. Figs. 1 and 3 present simulated phase structures of the numerical solutions and the corresponding numerical error at the terminal time with the interface thickness $\epsilon =1/16,1/32,1/64$, respectively. In both Figures, the three solutions on the top row are plotted on regular scale and the errors on the bottom are plotted on log scale. We clearly observe that as the interface thickness decreases, the transition zone gradually becomes narrower and the shape of the approximated region increasingly resembles the exact region. The inner phase structure depends on the intensity of fluctuations in the exact solution *u* and the phase-field function $\varphi$. Numerical errors are generally larger near the domain boundaries and in regions where the objective function changes rapidly, while in the interior and in smoothly varying regions, the errors are smaller.

### The case of varying diffusion coefficient

#### Example 2

In this example, we consider the following two-dimensional diffusion problem in the same circular domain and the flower-shaped domain used in Example 1, but with varying diffusion coefficient: for $0 \le t \le T$,

$$\begin{aligned} \left\{ \begin{array}{ll} \,u_t=\nabla \cdot \left( (x^2+y^2+3)\nabla u\right) +f(t,x,y), \   & (x,y)\in D\\ \,u(0,x,y) = \left( 2 x^2-4x\right) \left( 2 y^2-4y \right) , \ \ & (x, y) \in D, \end{array} \right. \end{aligned}$$

where

$$\begin{aligned} \begin{aligned} f(t,x,y) =&-e^{-\pi ^2 t}\Big (\pi ^2\left( 2 x^2-4x\right) \left( 2 y^2-4y\right) +\left( 4(x^2+y^2+3)+2x(4x-4)\right) \\&\cdot \left( 2 y^2-4y \right) +\left( 4(x^2+y^2+3)+2y(4y-4)\right) \left( 2 x^2-4x \right) \Big ), \end{aligned} \end{aligned}$$

**Table 2:** Numerical results on the solution solutions measured in the weighted $L^2$ and $H^1$ norms and corresponding convergence rates at the terminal time $T=0.5$ produced by the DDM in Example 2

$\epsilon$	$N_x\times N_y$	$N_T$	$\Vert u^{\epsilon }-u(t_n)\Vert _{L^2(D;\omega _{\epsilon })}$	CR	$\Vert u^{\epsilon }-u(t_n)\Vert _{H^1(D;\omega _{\epsilon })}$	CR
Approximation tests for circle domain						
1/8	$512\times 512$	512	4.4000e-03	-	4.4000e-03	-
1/16	$512\times 512$	512	1.1000e-03	2.00	1.2000e-03	1.87
1/32	$512\times 512$	512	2.8780e-04	1.93	3.2838e-04	1.87
1/64	$512\times 512$	512	7.5129e-05	1.94	1.6267e-04	1.01
Approximation tests for flower-shaped domain						
1/8	$512\times 512$	512	3.3000e-03	-	3.4000e-03	-
1/16	$512\times 512$	512	8.6685e-04	1.93	8.8274e-04	1.95
1/32	$512\times 512$	512	2.2363e-04	1.95	2.4955e-04	1.82
1/64	$512\times 512$	512	5.7174e-05	1.97	1.1470e-04	1.12

**Fig. 4:**
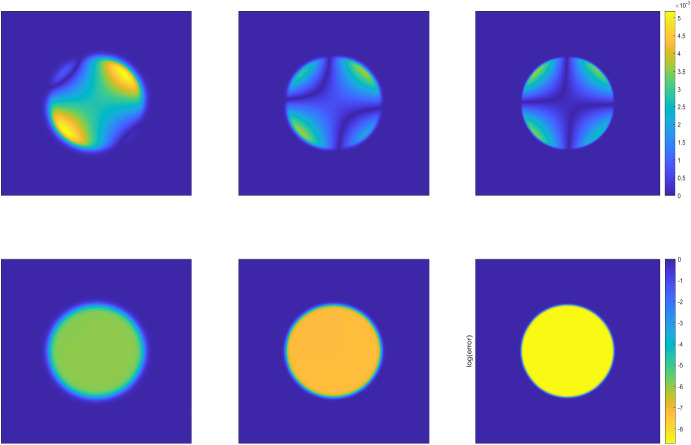
The phase structures of the numerical solutions (top row) and the corresponding numerical error (bottom row) at the terminal time $T=0.5$ produced by the DDM approach with interface thickness $\epsilon =1/16,1/32,1/64$ (from left to right) for Example 2 in the circular domain

**Fig. 5:**
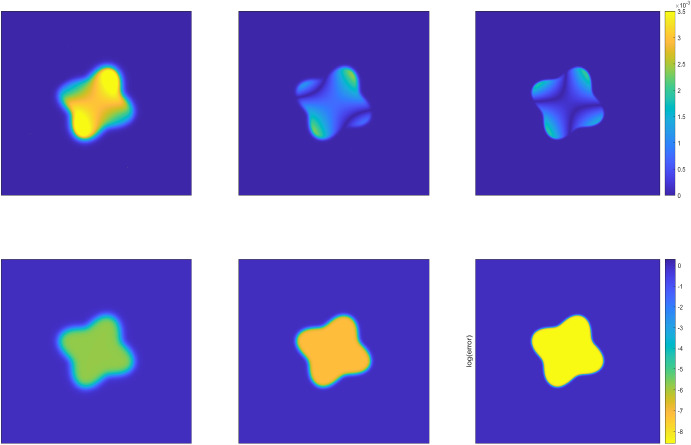
The phase structures of the numerical solutions (top row) and the corresponding numerical error (bottom row) at the terminal time $T=0.5$ produced by the DDM approach with interface thickness $\epsilon =1/16,1/32,1/64$ (from left to right) for Example 2 in the flower domain

In this case, the exact solution is given by

$$\begin{aligned} u(t,x,y)=e^{-\pi ^2 t}\left( 2 x^2-4x \right) \left( 2 y^2-4y \right) . \end{aligned}$$

Circular domain and flower-shaped domain defined in (49) and (50) are considered again. The Neumann boundary condition is correspondingly imposed and the terminal time is set to $T=0.5$.

We verify the approximation accuracy of DDM by fixing $N_T=512$ (i.e., $\Delta \tau = T/N_T=1/1024$) and the extended rectangular domain $\Omega =[-1/2,1/2]\times [-1/2,1/2]$ ($D \subset \Omega$) with uniform spatial meshes, and the corresponding mesh size is $h_x=h_y=1/512$. To guarantee the spatial mesh sizes and temporal step size are much finer than the interface thickness $\epsilon$, we set $\epsilon =1/8,1/16,1/32, 1/64$ respectively for approximation accuracy tests. Table 2 shows the solution errors measured in the weighted $L^2$ and $H^1$ norms, including the corresponding convergence rates. From the Table 2, we can conclude that the DDM method exhibits second-order convergence rate in the weighted $L^2$ norm, and first-order convergence rate in the weighted $H^1$ norm. The numerical results match very well with the error estimates derived in Theorems 6 and 7. Fixing the same spatial meshes and temporal partitions, the simulated phase structures of the numerical solutions and the corresponding numerical error at the terminal time are shown in the Fig. 4 and Fig. 5 with interface thickness $\epsilon =1/16,1/32,1/64$, respectively. In both Figures, the three solutions on the top row are plotted on regular scale and the errors on the bottom are plotted on log scale. It’s observed again that with the interface thickness decreasing, the transition zone gradually becomes narrower and narrower, and the shape of the approximated region resembles the original irregular domain better and better. Similar as the Example 1, the phase structure of the numerical solution mainly depends on the intensity of fluctuations in the exact solution *u* and the phase-field function $\varphi$. The numerical error tends to be more significant near the domain boundaries and in areas where the objective function changes rapidly, while that tends to be smaller in the interior of the domain due to the smoothness of the function.

## Conclusions

In this paper, we studied the convergence and error estimates of the diffuse domain method for solving a class of second-order parabolic equations with Neumann boundary conditions in general irregular domains. We successfully proved optimal error estimates of the diffuse domain solutions in the weighted $L^2$ and $H^1$ norms. Some numerical examples are also presented to verify the derived theoretical results. The numerical method and corresponding error analysis framework developed here also naturally enable us to further investigate adaptive finite element methods [9, 10, 16] for solving PDEs in regions with corner points with solid theoretical support.

## References

[1] Abels, H.: On a diffuse interface model for two-phase flows of viscous, incompressible fluids with matched densities. Arch. Ration. Mech. Anal. **194**(2), 463–506 (2009). 10.1007/s00205-008-0160-2 10.1007/s00205-024-02020-9 PMC11371890 39239088

[2] Abels, H., Lengeler, D.: On sharp interface limits for diffuse interface models for two-phase flows. Interfaces Free Bound. **16**(3), 395–418 (2014). 10.4171/IFB/324

[3] Adams, R.A., Fournier, J.J.F.: Sobolev Spaces, volume 140 of Pure and Applied Mathematics (Amsterdam). Elsevier/Academic Press, Amsterdam and Boston, 2nd edition (2003). ISBN 0-12-044143-8

[4] Aland, S., Lowengrub, J., Voigt, A.: Two-phase flow in complex geometries: a diffuse domain approach. CMES Comput. Model. Eng. Sci. **57**(1), 77–107 (2010) 21918638 PMC3171464

[5] Allen, S., Cahn, J.: A microscopic theory for antiphase domain boundary motion and its application to antiphase domain coarsening. Acta Metall. **27**, 1085–1095 (1979). 10.1016/0001-6160(79)90196-2

[6] Anderson, D.M., McFadden, G.B., Wheeler, A.A.: Diffuse-interface methods in fluid mechanics. Annu. Rev. Fluid Mech. **30**(1), 139–165 (1998). 10.1146/annurev.fluid.30.1.139

[7] Barua, A.K., Chew, R., Li, S., Lowengrub, J., Münch, A., Wagner, B.: Sharp-interface problem of the Ohta-Kawasaki model for symmetric diblock copolymers. J. Comput. Phys. **481**, 112032 (2023). 10.1016/j.jcp.2023.112032

[8] Bedrossian, J., von Brecht, J.H., Zhu, S., Sifakis, E., Teran, J.M.: A second order virtual node method for elliptic problems with interfaces and irregular domains. J. Comput. Phys. **229**(18), 6405–6426 (2010). 10.1016/j.jcp.2010.05.002

[9] Bieterman, M., Babuška, I.: The finite element method for parabolic equations. I. A posteriori error estimation. Numer. Math. **40**(3), 339–371 (1982). 10.1007/BF01396451

[10] Binev, P., Dahmen, W., DeVore, R.: Adaptive finite element methods with convergence rates. Numer. Math. **97**(2), 219–268 (2004). 10.1007/s00211-003-0492-7

[11] Brannick, J., Liu, C., Qian, T., Sun, H.: Diffuse interface methods for multiple phase materials: an energetic variational approach. Numer. Math. Theory Methods Appl. **8**(2), 220–236 (2015). 10.4208/nmtma.2015.w12si

[12] Bueno-Orovio, A., Pérez-García, V.M., Fenton, F.H.: Spectral methods for partial differential equations in irregular domains: the spectral smoothed boundary method. SIAM J. Sci. Comput. **28**(3), 886–900 (2006). 10.1137/040607575

[13] Bukač, M., Muha, B., Salgado, A.J.: Analysis of a diffuse interface method for the Stokes-Darcy coupled problem. ESAIM Math. Model. Numer. Anal. **57**(5), 2623–2658 (2023). 10.1051/m2an/2023062

[14] Burger, M., Elvetun, O.L., Schlottbom, M.: Diffuse interface methods for inverse problems: case study for an elliptic Cauchy problem. Inverse Prob. **31**(12), 125002 (2015). 10.1088/0266-5611/31/12/125002

[15] Burger, M., Elvetun, O.L., Schlottbom, M.: Analysis of the diffuse domain method for second order elliptic boundary value problems. Found. Comput. Math. **17**(3), 627–674 (2017). 10.1007/s10208-015-9292-6

[16] Ciarlet, P.G.: The Finite Element Method for Elliptic Problems, volume 40 of *Classics in Applied Mathematics*. Society for Industrial and Applied Mathematics (SIAM), Philadelphia, PA (2002). ISBN 978-0-89871-514-9. 10.1137/1.9780898719208

[17] Dolbow, J., Harari, I.: An efficient finite element method for embedded interface problems. Internat. J. Numer. Methods Engrg. **78**(2), 229–252 (2009). 10.1002/nme.2486

[18] Du, Q., Feng, X.: The phase field method for geometric moving interfaces and their numerical approximations. In: Handbook of Numerical Analysis, Volume 21: Geometric Partial Differential Equations – Part I, pp. 425–508. Elsevier / North-Holland, Amsterdam (2020). 10.1016/bs.hna.2019.05.001

[19] Elliott, C.M., Stinner, B., Styles, V., Welford, R.: Numerical computation of advection and diffusion on evolving diffuse interfaces. IMA J. Numer. Anal. **31**(3), 786–812 (2011). 10.1093/imanum/drq005

[20] Feireisl, E., Petzeltová, H., Rocca, E., Schimperna, G.: Analysis of a phase-field model for two-phase compressible fluids. Math. Models Methods Appl. Sci. **20**(7), 1129–1160 (2010). 10.1142/S0218202510004544

[21] Franz, S., Gärtner, R., Roos, H.-G., Voigt, A.: A note on the convergence analysis of a diffuse-domain approach. Comput. Methods Appl. Math. **12**(2), 153–167 (2012). 10.2478/cmam-2012-0017

[22] Fries, T.-P., Belytschko, T.: The extended/generalized finite element method: an overview of the method and its applications. Internat. J. Numer. Methods Engrg. **84**(3), 253–304 (2010). 10.1002/nme.2914

[23] Frigeri, S., Grasselli, M., Rocca, E.: A diffuse interface model for two-phase incompressible flows with non-local interactions and non-constant mobility. Nonlinearity **28**(5), 1257–1293 (2015). 10.1088/0951-7715/28/5/1257

[24] Griffiths, D.F.: The Mathematical Basis of Finite Element Methods: With Applications to Partial Differential Equations. Clarendon Press, Oxford (1984)

[25] Guo, Z., Fei, Yu., Lin, P., Wise, S., Lowengrub, J.: A diffuse domain method for two-phase flows with large density ratio in complex geometries. J. Fluid Mech. **907**, A38 (2021). 10.1017/jfm.2020.790

[26] Hellrung, J.L., Jr., Wang, L., Sifakis, E., Teran, J.M.: A second order virtual node method for elliptic problems with interfaces and irregular domains in three dimensions. J. Comput. Phys. **231**(4), 2015–2048 (2012). 10.1016/j.jcp.2011.11.023

[27] Jerg, K.I., Austermühl, R.P., Roth, K., Sundrup, J.G., Kanschat, G., Hesser, J.W., Wittmayer, L.: Diffuse domain method for needle insertion simulations. Int. J. Numer. Methods Biomed. Eng. **36**(9), e3377 (2020). 10.1002/cnm.3377 10.1002/cnm.3377 32562345

[28] Kannenberg, T., Schöller, L., Prahs, A., Schneider, D., Nestler, B.: Microstructure evolution accounting for crystal plasticity in the context of the multiphase-field method. Comput. Mech. **74**(1), 67–84 (2024). 10.1007/s00466-023-02423-7

[29] Kockelkoren, J., Levine, H., Rappel, W.-J.: Computational approach for modeling intra- and extracellular dynamics. Phys. Rev. E **68**, 037702 (2003). 10.1103/PhysRevE.68.037702 10.1103/PhysRevE.68.037702 14524934

[30] Lervåg, K.Y., Lowengrub, J.: Analysis of the diffuse-domain method for solving PDEs in complex geometries. Commun. Math. Sci. **13**(6), 1473–1500 (2015). 10.4310/CMS.2015.v13.n6.a6 10.4310/cms.2009.v7.n1.a4 PMC3097555 21603084

[31] LeVeque, R.J., Li, Z.L.: The immersed interface method for elliptic equations with discontinuous coefficients and singular sources. SIAM J. Numer. Anal. **32**(5), 1704 (1995). 10.1137/0732076

[32] Li, J.J., Feng, X.F.: Higher-order finite difference scheme for solving one-dimensional elliptic equations with discontinuous coefficient and singular sources. Commun. Appl. Math. Comput. **29**(4), 503–513 (2015)

[33] Li, X., Lowengrub, J., Rätz, A., Voigt, A.: Solving PDEs in complex geometries: a diffuse domain approach. Commun. Math. Sci. **7**(1), 81–107 (2009). 10.4310/cms.2009.v7.n1.a4 21603084 10.4310/cms.2009.v7.n1.a4 PMC3097555

[34] Li, Z., Ito, K.: The Immersed Interface Method: Numerical Solutions of PDEs Involving Interfaces and Irregular Domains, volume 33 of Frontiers in Applied Mathematics. Society for Industrial and Applied Mathematics (SIAM), Philadelphia, PA (2006). 10.1137/1.9780898717464

[35] Liu, X., Chai, Z., Zhan, C., Shi, B., Zhang, W.: A diffuse-domain phase-field lattice Boltzmann method for two-phase flows in complex geometries. Multiscale Model. Simul. **20**(4), 1411–1436 (2022). 10.1137/22M1475120

[36] Nguyen, L.H., Stoter, S.K.F., Ruess, M., Sanchez Uribe, M.A., Schillinger, D.: The diffuse Nitsche method: dirichlet constraints on phase-field boundaries. Internat. J. Numer. Methods Engrg. **113**(4), 601–633 (2018). 10.1002/nme.5628

[37] Prahs, A., Schöller, L., Schwab, F.K., Schneider, D., Böhlke, T., Nestler, B.: A multiphase-field approach to small strain crystal plasticity accounting for balance equations on singular surfaces. Comput. Mech. **73**(4), 773–794 (2024). 10.1007/s00466-023-02389-6

[38] Rätz, A., Voigt, A.: PDE’s on surfaces–a diffuse interface approach. Commun. Math. Sci. **4**(3), 575–590 (2006). 10.4310/cms.2006.v4.n3.a5

[39] Schlottbom, M.: Error analysis of a diffuse interface method for elliptic problems with Dirichlet boundary conditions. Appl. Numer. Math. **109**, 109–122 (2016). 10.1016/j.apnum.2016.06.005

[40] Stoter, S.K.F., Müller, P., Cicalese, L., Tuveri, M., Schillinger, D., Hughes, T.J.R.: A diffuse interface method for the Navier-Stokes/Darcy equations: perfusion profile for a patient-specific human liver based on MRI scans. Comput. Methods Appl. Mech. Engrg. , 70–102 (2017). 10.1016/j.cma.2017.04.002

[41] Wang, C., Chertock, A., Cui, S., Kurganov, A., Zhang, Z.: A diffuse-domain-based numerical method for a chemotaxis-fluid model. Math. Models Methods Appl. Sci. **33**(2), 341–375 (2023). 10.1142/S0218202523500094

[42] West, J.R., Adler, M.C., Lele, S.K.: A high-order, localized-artificial-diffusivity method for Eulerian simulation of multi-material elastic-plastic deformation with strain hardening. J. Comput. Phys. **514**, 113205 (2024). 10.1016/j.jcp.2024.113205

[43] Xiao, W., Liu, K., Lowengrub, J., Li, S., Zhao, M.: Three-dimensional numerical study on wrinkling of vesicles in elongation flow based on the immersed boundary method. Phys. Rev. E **107**(3), 035103 (2023). 10.1103/physreve.107.035103 37072945 10.1103/PhysRevE.107.035103

[44] Yang, J., Mao, S., He, X., Yang, X., He, Y.: A diffuse interface model and semi-implicit energy stable finite element method for two-phase magnetohydrodynamic flows. Comput. Methods Appl. Mech. Engrg. **356**, 435–464 (2019). 10.1016/j.cma.2019.07.022

[45] Zhao, S., Wei, G.W.: Matched interface and boundary (MIB) for the implementation of boundary conditions in high-order central finite differences. Internat. J. Numer. Methods Engrg. **77**(12), 1690–1730 (2009). 10.1002/nme.2473 10.1002/nme.2473 PMC2871773 20485574

